# Contribution of North Atlantic temperature variability to El Niño cyclicity as revealed by spectral causality estimates

**DOI:** 10.1038/s41598-026-48274-z

**Published:** 2026-04-20

**Authors:** Igor I. Mokhov, Dmitry A. Smirnov

**Affiliations:** 1 Obukhov Institute of Atmospheric Physics of the Russian Academy of Sciences, 3 Pyzhevsky Per., Moscow, Russia 603950; 2 Department of Physics, Lomonosov Moscow State University, Leninskiye Gory, Moscow, Russia 119991; 3 Saratov Branch of Kotelnikov Institute of Radioengineering and Electronics of the Russian Academy of Sciences, 38 Zelyonaya St., Saratov, Russia 410019

**Keywords:** El Niño/Southern Oscillation, Intradecadal North Atlantic temperature variability, Climate indices, Time series analysis, Directional couplings, Spectral causal effects, Climate sciences, Ocean sciences

## Abstract

Spectral effects of directional (causal) couplings between El Niño – Southern Oscillation (ENSO) and intradecadal North Atlantic temperature variability (NATV) are estimated from monthly time series of their indices over the period of 1870 – 2022 on the basis of empirical autoregressive models. For any of the three ENSO indices (Niño-3, Niño-3,4 and Niño-4), a bidirectional coupling between the ENSO and the NATV (i.e. both the influence of the ENSO on the NATV and that in the opposite direction) is detected. According to the models, removal of the coupling in any of the two directions leads to disappearance of the spectral peak on the periods of 3–5 years for the NATV and at least to a strong blurring of that peak for the ENSO. The spectral causal effect (i.e. power spectrum changes under the respective coupling removal) of the NATV on the eastern equatorial Pacific region Niño-3 is found to be the strongest one among the three ENSO regions, while that effect on the most western region Niño-4 is the smallest one.

**Supplementary Information:**

The online version contains supplementary material available at 10.1038/s41598-026-48274-z.

## Introduction

In modeling global climate processes and predicting climate changes, it is important to adequately describe and quantitatively estimate couplings (interactions) between the key global and regional processes which are often defined as certain “modes of variability” via multiple existing approaches. In particular, an important role in the global and regional climate variability is played by the processes related to El Niño – Southern Oscillation (ENSO) in the equatorial latitudes of the Pacific Ocean, Atlantic Multidecadal Oscillation (AMO) in the North Atlantic and other key quasi-cyclic processes^1^. Couplings between ENSO and North Atlantic processes have been studied from observational data with diverse methods (e.g.^2–17^) often with emphasis on the statistical significance of the obtained estimates. Thus, the influence of AMO on ENSO is detected and discussed in^2,6–10,14,15^, the opposite one is found in^3,4^, while a bidirectional coupling between the two regions is shown in^11,12^.

Along with the detection of couplings between key climate processes, it is important to assess their role in the dynamics of the processes under investigation. A coupling may well be statistically reliably detected due to a sufficient amount and quality of data but non-important, e.g., if the changes in the observed dynamics under its disappearance or strong decrease are small. In studies of quasi-cyclic processes like ENSO or AMO, an especial interest is attracted to the evaluation of the role of couplings in forming spectral features of those processes, e.g., their intradecadal, decadal and interdecadal variability. Thus, pronounced spectral peaks characterize the intradecadal (or interannual) variability of ENSO in the interval from 2 to 8 years, e.g.^18–25^, which is a key component. North Atlantic temperature variability (NATV, i.e. the ocean surface temperatures over the region 0 to 70^o^ N) exhibits strong decadal and multidecadal components, e.g.^26–30^. AMO represents the dominant mode of the NATV. However, the NATV periodicity in the interval from 2 to 3 years has also been detected as significant, e.g.^31^ Therefore, one may well expect the presence of strong directional couplings between the intradecadal components of the two processes. Then, an important question is whether such couplings are dynamically important, i.e. whether they strongly contribute to producing and maintaining the intradecadal spectral components of the ENSO and NATV. This question is addressed here via the “directional spectral approaches” which seem to be promising in climate data analysis.

Several spectral causality measures have been developed to quantify the role of directional couplings^32–51^. Among them, spectral causal effects suggested within the framework of dynamical causal effects (DCE)^49–51^ directly quantify the changes of spectral properties of the observed dynamics under hypothetical removal of coupling in a given direction. Such measures seem to be the most appropriate ones to answer the above question and should be able to extract new information from climate time series at hand. Therefore, they are the main tool used in this work. Below, we evaluate several spectral causal effects via fitting empirical autoregressive (AR) models to observed data. In addition, we estimate a short-term causal effect reflected by Granger causality^52^ and a long-term causal effect on stationary variance^12^. A brief preliminary result of such estimation for a single ENSO index Niño-3,4 without robustness tests is reported in^53^, while here we systematically extend it to three different ENSO indices (regions), different temporal intervals and various parameters of the data and methods.

## Results

The optimal AR models obtained from time series under study and their characteristics are described below as an intermediate result of the work. Then, various directional coupling quantifiers between the processes under study including spectral causal effects computed on the basis of those optimal AR models are presented.

**Fig. 1:**
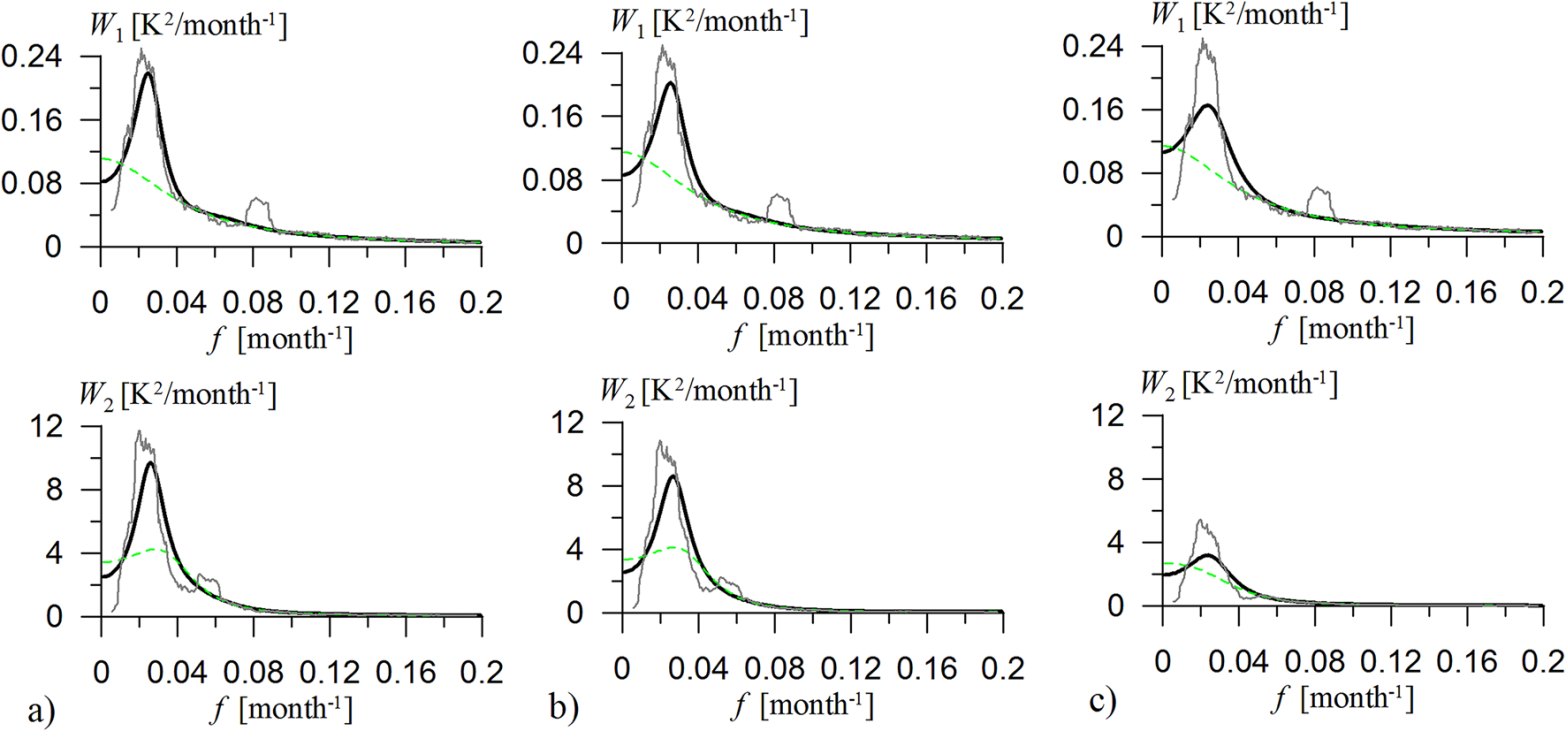
Power spectral densities for the indices *x*_1_ (NATV, top panels) and *x*_2_ (ENSO, bottom panels) according to the full AR models (1) (black solid lines) and to the uncoupled AR models (green dashed lines). Thin grey lines show smoothed periodograms of the time series under analysis (details are given in the SI): a) *x*_2_ is Niño-3; b) *x*_2_ is Niño-3,4; c) *x*_2_ is Niño-4.

**Models.** The optimal AR models of the NATV and the ENSO for the period 1870 – 2022 are obtained from the time series high-pass filtered via subtraction of the 5-year moving average. This filter retains variability with periods of 7–8 years and less, while strongly suppresses variability with periods of 9–10 years and greater, see Sec. S1.2 and Fig. S4 in Supplementary Information (SI). Moving window lengths ranging from 3 to 10 years are also checked as commented in the end of this Section. For any ENSO index used as the variable $x_{2}$ (while $x_{1}$ stays for NATV, see “Data”), the optimal AR model obtained from data (SI, Sec.S1.3) reads

1 $${x_{i} (t) = \sum\limits_{k = 1}^{{d_{i} }} {a_{i,k} x_{i} (t - k)} + \sum\limits_{i = 1}^{{d_{j \to i} }} {b_{i,k} x_{j} (t - k)} + \eta_{i} (t),\;\;\;i,j = 1,2,\;i \ne j,}$$

with the dimensions (orders) of individual components $d_{1} = 1$ for the NATV and $d_{2} = 6$ for the ENSO. The dimensions of the coupling components depend on the ENSO index. The influence of the ENSO on the NATV is described by $d_{2 \to 1} = 5$ terms for the index Niño-3 used as $x_{2}$ and by $d_{2 \to 1} = 3$ terms for Niño-3,4 and Niño-4. The influence of the NATV on the ENSO is described by $d_{1 \to 2} = 12$ terms for the indices Niño-3 and Niño-3,4 and by $d_{1 \to 2} = 6$ terms for Niño-4. These quite different values of model dimensions for the two processes are obtained adaptively from the data (see “Methods”) and assumed to be due to different dynamical properties of these processes. The estimates of the model coefficients are presented for the index Niño-3,4 used as $x_{2}$ in Table S2 of SI. The model noises (prediction errors) ${\eta_{1} }$ and ${\;\eta_{2} }$ do not exhibit considerable deviations from the conditions of delta-correlatedness and Gaussianity for all ENSO indices (see Fig. S10 in SI), their cross-correlation coefficient does not significantly differ from zero, e.g., it equals $0.02 \pm 0.05$ for the index Niño-3,4 used as $x_{2}$. As for the frequency domain, the model (1) reproduces power spectra of both processes under study reasonably well (Fig. 1a-c), especially for the indices Niño-3 (Fig. 1,a) and Niño-3,4 (Fig. 1,b). All those features justify applicability of the optimal AR models (1) to the evaluation of the spectral causal effects and other coupling quantifiers below.

**Coupling detection.** Short-term DCEs represented by the Granger causality measure (see “Methods”) reveal a bidirectional coupling between the intradecadal components of the ENSO and the NATV, which manifests itself most strongly for the index Niño-3 and most weakly for the index Niño-4 (see Sec. S1.3 in SI). This conclusion is highly statistically significant at *p*-levels of the Fisher *F*-test less (and sometimes much less) than 10^–5^ as described in SI (Table S4).

**“Average” dynamical role.** Long-term DCEs with respect to variance (6) according to the AR models (1) are following: $C_{2 \to 1}\approx {25\%}$, $C_{1 \to 2}$ ranges from 28% for the index Niño-3 to 12% for the index Niño-4 (Table 1). Thus, the models evidence that the variances of both Atlantic and Pacific processes rise due to arousal of the detected bidirectional coupling approximately by one quarter (only the variance of the index Niño-4 rises just by 1/8). Such contribution of the coupling to the long-term properties of the observed processes is quite considerable, so the couplings are also “dynamically” (rather than only statistically) significant. These effects of couplings are the strongest for the index Niño-3 and considerably smaller for the index Niño-4.

**Table 1:** Estimates of directional coupling quantifiers for three ENSO indices for the period 1870 – 2022 which show the effects of “switching the coupling on”: relative change of stationary variance $C_{j \to i}$, maximal and minimum relative changes of power spectral density ${S_{j \to i}^{\max } }$ and ${S_{j \to i}^{\min } }$, change of spectral peak sharpness ${R_{j \to i} }$ shown as ${R_{i} - R_{{i\left| {b_{i,k} = 0} \right.}} }$ (see “Methods”).

Coupling quantifiers	${I_{N3} }$	${I_{N34} }$	${I_{N4} }$
$C_{2 \to 1}$	0.26	0.24	0.23
${C_{1 \to 2} }$	0.28	0.26	0.12
${S_{2 \to 1}^{\max } }$	1.6	1.4	1.0
${S_{1 \to 2}^{\max } }$	1.3	1.1	0.6
${S_{2 \to 1}^{\min } }$	− 0.3	− 0.3	− 0.1
${S_{1 \to 2}^{\min } }$	− 0.3	− 0.2	− 0.3
${R_{2 \to 1} }$	1.7–0.0	1.3–0.0	0.6–0.0
${R_{1 \to 2} }$	2.9–0.2	2.3–0.2	0.6–0.0

**Spectral causal effects.** The role of the directional couplings in the dynamics of the ENSO and the NATV is even more important according to the estimates of their spectral effects [see Eq. (3) and (4)]. Figure 1 presents the power spectral density of each process ${x_{i} }$ for the full AR model (1) and for the same AR model with zero coupling coefficients. The full model reproduces reasonably well the observed spectra with their clear peak in the interval of 2.5–5 years (Fig. 1, thin grey lines). The maxima of the model spectra occur at the periods of 38 months for the NATV (sharpness of the peak is ${R_{1} = }1.3$, Fig. 1, top panels, solid black lines) and 37 months for the ENSO (sharpness ${R_{2} = 2}.3$, Fig. 1, bottom panels, solid black lines). Such sharpness means that the spectral peaks exceed the zero-frequency power spectral density 2.3 and 3.3 times, respectively (see “Methods”). Dynamically, it implies that one sees irregularly perturbed oscillations with a period of about 3 years in both time series, especially in ${x_{2} }$ (ENSO). Under zeroing the couplings, the spectral peak for ${x_{1} }$ (NATV) disappears (sharpness ${R_{{1\left| {b_{1,k} = 0} \right.}} = 0}$, Fig. 1, top panels, green dashed lines), while that peak for ${x_{2} }$ (ENSO) strongly blurs (${R_{{2\left| {b_{2,k} = 0} \right.}} }$ ranges from 0.2 to 0, Fig. 1, bottom panels, green dashed lines). It means that “switching the bidirectional coupling on” in the AR model (1) under other equal conditions leads to arousal of discernible oscillations with a period of about 3 years in both time series. Table 1 presents the corresponding DCEs with respect to the spectral maximum sharpness (4) as the differences between the values of the sharpness parameter for the estimated and zero couplings: ${R_{2 \to 1} }$ ranges from 1.7 (for the index Niño-3) to 0.6 (for Niño-4) and ${R_{1 \to 2} }$ ranges from 2.7 (for Niño-3, Fig. 1a) to 0.6 (for Niño-4, Fig. 1c).

**Fig. 2:**
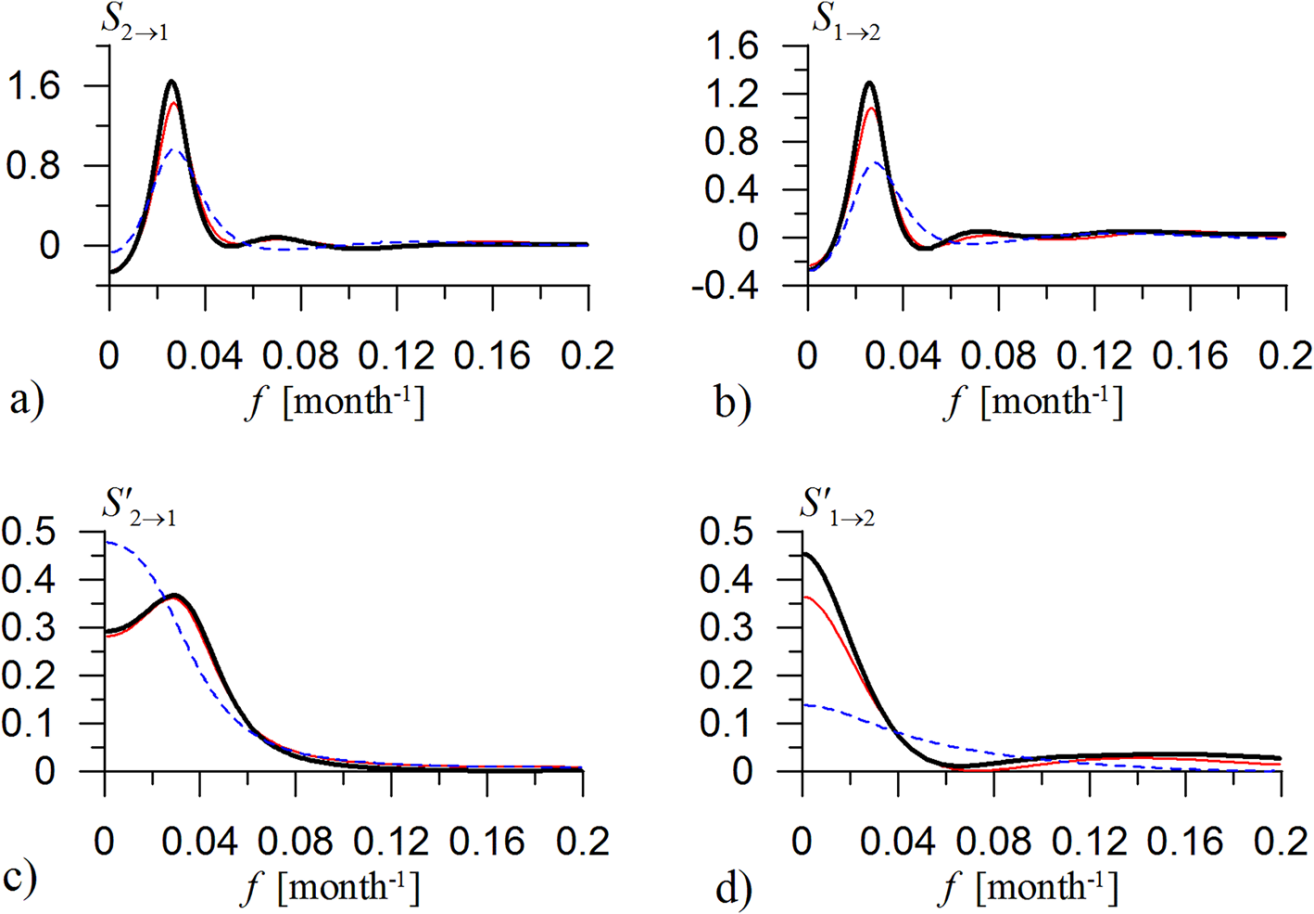
Spectral DCEs (3) for the full AR models (1) (**a**, **b**) and spectral DCEs (5) for the unidirectionally coupled AR models (**c**, **d)** obtained for three ENSO indices: Niño-3 (thick black lines), Niño-3,4 (thin red), and Niño-4 (dashed blue). Left panels show the DCEs in the direction ENSO → NATV (**a**, c), right panels in the direction NATV → ENSO (**b**, **d**).

The relative spectral DCEs (3) in both directions are maximal in the interval of periods of 2.5–5 years (Fig. 2a,b) and close to each other, usually exceeding unity which corresponds to the increase of the power spectral density more than twice due to switching the coupling on. Under the usage of the index Niño-3, one gets ${S_{2 \to 1}^{\max } } = 1.6$ (i.e. the maximal rise of the power spectral density due to arousal of the coupling is 2.6 times) and ${S_{1 \to 2}^{\max } } = 1.3$. For low frequencies, the spectral DCEs are negative, e.g., one gets ${S_{2 \to 1}^{\min } }={S_{1 \to 2}^{\min } }= -0.3$ at zero frequency under the usage of the index Niño-3, i.e. switching the coupling on decreases the power spectral density almost by one-third. Due to such decrease of the power spectral density near zero frequency and its rise in the interval of periods of 2.5–5 years, the spectral peaks under study either arise or get much sharper. The results are quite close for the index Niño-3,4, while for the index Niño-4 the spectral DCEs are weaker but the qualitative conclusion remains the same. It is interesting to note that the influence NATV → ENSO is in general not considerably weaker and according to some quantifiers (${R_{1 \to 2} }$, ${C_{1 \to 2} }$) it is even stronger than the opposite influence. This observation is physically interpretable, being consistent with the Atlantic capacitor mechanism^16^ and triggering of the El Niño events by the north tropical Atlantic temperature decrease via subsequent changes in the Atlantic and Pacific intertropical convergence zones.^9^

As for the spectral role of each coupling in its unidirectional version (i.e. under zero coupling in the opposite direction and other equal conditions), it is not as large and does not lead to arousal of the peaks under study (Fig. 2c,d). In particular, consider the power spectral density of the signal ${x_{2} }$ (ENSO) under the removal of its outgoing coupling ${2 \to 1}$ (ENSO → NATV) and retaining its incoming coupling ${1 \to 2}$ (NATV → ENSO). Since the index ${x_{1} }$ (NATV) is a red noise, the unidirectional coupling NATV → ENSO (Fig. 2d) cannot enhance in this case sharpness of the spectral peak of ${x_{2} }$ (ENSO) whose power spectrum remains as blurred as that for the individual AR model of ${x_{2} }$ in Fig. 1. Analogously, if one removes the coupling ${1 \to 2}$ (NATV → ENSO) and retains only the coupling ${2 \to 1}$ (ENSO → NATV), the spectral peak for ${x_{1} }$(NATV) is absent even for that nonzero coupling from ${x_{2} }$ (ENSO).

Spectral causal effects in Table 1 are presented via their point estimates without a special evaluation of statistical significance. However, the directional couplings themselves are found above to be non-zero with high confidence, so the respective spectral causal effects are non-zero with the same confidence. As for the shapes of their plots in Fig. 2 and their extremal values, the respective conclusions require a separate evaluation of their confidence, but a rigorous statistical procedure for that does not exist. Still, considerable variations of different parameters of the data and method in this work (see below) almost do not change the shapes of these plots and the frequency of spectral peaks. Moreover, two kinds of surrogate data tests confirm high significance of the obtained nonzero spectral causal effects (Sec.S2.7 in SI). All that may serve as a reasonable substitute for a direct statistical significance test.

To summarize, all the obtained results evidence that within the AR model framework used here, the bidirectional coupling between the two processes under study (Pacific and Atlantic) is a necessary condition for the existence of a clear spectral peak in the range of periods of 2.5–5 years. According to all estimated coupling quantifiers for each of the two directions, the strongest coupling between the NATV and the ENSO is obtained for the eastern region Niño-3 (the closest one to Atlantic), while the coupling is somewhat weaker for the region Niño-3,4 (located more to the west) and considerably weaker for the region Niño-4 (the most distant from Atlantic). The difference between the coupling estimates for the indices Niño-3 and Niño-4 can be considered as statistically significant (Sec. S3 in SI).

These conclusions are quite robust to variations of parameters of the data and methods as demonstrated in SI (Secs. S2 and S4) where details of the estimation results and the robustness evaluation are presented. In particular, an important part of the analysis procedure is the high-pass filter used to extract faster components of the processes. Variations of its moving window length in the range from 3 to 7 years lead to quite moderate quantitative changes of the spectral causal effects (SI, Sec. S2.5) retaining qualitative conclusion about the spectral role of the bidirectional coupling. Even the 10-yr moving window retains this conclusion though it makes the spectral effects considerably weaker. Thus, the results are quite robust to the filter parameter variations as soon as the components under study remain in the intradecadal range. Other kinds of filters deserve to be studied systematically along with sensitivity of the entire method to the filter kind. However, the presented choice already provides quite natural separation of the faster and slower components and does not change the phase spectra of the signals under study, so it seems to be sufficiently well justified for obtaining the first reasonable guess of the spectral causal effects of interest.

The above results are obtained for optimal AR models. However, variations of model dimensions in a wide range (checking various local minima of significance level estimate, see Sec.S2.1 in SI) do not change the results qualitatively, only the decrease of the “coupling term dimension” to ${d_{{{1} \to {2}}} = 1}$ leads to disappearance of strong spectral causal effects. It evidences that the achieved conclusion of this work is robust with respect to the AR model structure.

Robustness of the results across time is shown in Sec. S4, where the entire estimation procedure is repeated for the two halves of the observation period: 1870–1945 and 1946–2022. All conclusions about significant effects of the directional couplings appear to be qualitatively the same for both halves and for the entire period. Some quantitative changes between the two halves lead to an observation that the quantifiers of directional couplings between ENSO and NATV rise with time for the index Niño-3 and decrease with time for Niño-4 (with “mixed” conclusions for the “mixed” index Niño-3,4). Still, reliability of the latter conclusion about temporal evolution of the coupling quantifiers deserves a special study.

We repeat that all conclusions are made here within the framework of linear stationary AR models (1). Gaussianity and delta-corrlelatedness of the model residuals (SI, Fig.S10) justify this model class as an appropriate choice (see “Methods”). However, nonlinear and/or periodically non-stationary AR models deserve to be studied to find out whether some complicated details of the data under study may be taken into account and to refine the spectral causal effect estimates.

## Discussion and conclusions

A bidirectional coupling between the North Atlantic temperature variations and El Niño phenomena is revealed in this work from time series of their indices over the period 1870 – 2022 within the stationary linear AR modeling framework on the basis of the short-term causal effects (one-month-ahead prediction improvements) for three different ENSO indices. According to the obtained empirical AR models, the long-term causal effects with respect to variance show that the variance of each process rises due to the arousal of coupling from another process by about one-quarter. It evidences a considerable dynamical role of both directional couplings.

The main point of interest here is the spectral changes in the ENSO and the NATV in response to removal (insertion) of the couplings between them from (into) the AR models. The obtained estimates of the spectral causal effects for the periods range of 2.5–5 years are quite large: the power spectral density of both processes at those periods rises twice and even more than twice due to the insertion of the estimated bidirectional coupling into the AR models. Moreover, sharpness of these peaks increases essentially. So, within the AR model framework used here, the bidirectional coupling is a necessary condition for the existence of a clear spectral peak in the interval of periods of 2.5–5 years for both processes.

Analyses of three different ENSO indices reveal that the spectral DCEs obtained with the index *I*_*N3*_ (i.e. for the region Niño-3 which is the closest one to Atlantic) are greater than those for the indices *I*_*N34*_ and *I*_*N4*_, while those for the index *I*_*N4*_ (for the region Niño-4 most distant from Atlantic) are the smallest ones. Thus, according to the data over the period 1870 – 2022, the spectral role of the bidirectional coupling between the NATV and the ENSO is strongest for the index *I*_*N3*_ which characterizes the canonical El Niño phenomena.

All these conclusions are robust to variations of different parameters of the methods and data used including AR model dimensions and cut-off frequency of the high-pass filter. Moreover, the estimation results remain qualitatively the same for the shorter periods 1870–1945 and 1946–2022. Moderate quantitative differences imply that the directional couplings between ENSO and NATV rise with time for the region Niño-3 and decreases for Niño-4.

The methods used have some limitations implying that further studies of the problem may be appropriate. Thus, nonlinear and/or non-stationary AR models may be fitted to data to search for more accurate description of the observed dynamics. A special procedure for testing statistical significance of the spectral causal effects could provide more definite estimates of the reliability of various spectral conclusions. A systematic investigation of how the spectral causal effect estimates depend on the type of the high-pass filter could prompt the best filter choice for this and other problems of multivariate time series analysis. All such improvements may provide further details in addition to the obtained conclusions which still seem to be sufficiently well argued at the present stage.

It is worth mentioning that the bidirectional coupling in the obtained AR models plays a crucial role in producing the leading interannual periodicity of ENSO. This is probably the most interesting and unexpected implication of this work which appears to be consistent with the result of^16^ where the Atlantic capacitor mechanism is suggested to explain the biennial component of ENSO, but the idea could seemingly be extended to other frequency bands. The influence NATV → ENSO is explained in^9,16^ as being due to triggering of the El Niño events by the north tropical Atlantic via the Atlantic and Pacific intertropical convergence zones, which equally applies to the present work where the influence NATV → ENSO is also found to be quite significant and sometimes even stronger than the opposite one.

Finally, we note that applications of spectral causality quantifiers to climate data are almost not encountered in the literature. The present work illustrates that such methods, both well established^32–41^ and newly developed^48–52^, may well give substantial meaningful information about couplings between climate processes in addition to previously used directional coupling characteristics, e.g.^54–66^. In particular, in comparison with the well established Granger causality^52,55^ and transfer entropy-like^56,59^ approaches, spectral causal quantifiers may provide insights into the role of directional couplings in producing spectral peaks (i.e. considerable oscillatory components in the data under study) and determining their basic frequencies. Thereby, the presented results should motivate researchers to use much wider (and further improve) various spectral causality quantifiers in climate studies.

## Data and methods

*Data.* We have analyzed monthly data for ENSO and AMO indices over the time interval 1870 – 2022. El Niño phenomena are characterized by the sea surface temperature (SST) of the equatorial Pacific regions Niño-3 (5^o^ N – 5^o^ S, 150° – 90° W), Niño-3,4 (5^o^ N – 5^o^ S, 170° – 120° W) and Niño-4 (5^o^ N – 5^o^ S, 160° E – 150° W) according to the HadISST data^67^. Those SST data are taken from collections of measurements from ships, drifting and moored buoys and satellite data. The sea ice data are taken from a variety of sources including digitized sea ice charts and passive microwave retrievals. SSTs are reconstructed using a two stage reduced-space optimal interpolation procedure, while SSTs near sea ice are estimated using statistical relationships between SST and sea ice concentration. As an AMO index, we have mainly used the index *I*_*A*_^68,69^ which is the North Atlantic SST (0 – 70^o^ N). For comparison, the SI presents the results for the index *I*_*A2*_ (instead of *I*_*A*_) according to the ERSSTv5 data^70^ for the SST of the North Atlantic region 0–60^o^ N. The data of^68–70^ also involve complex reconstruction techniques as mentioned above for the data of^67^. The previous works^11,12^ estimated the short-term DCEs for the indices *I*_*A*_ and *I*_*N34*_ over the period 1870 – 2013. Here, we have analyzed the time series of the high-pass filtered AMO index *I*_*A*_ and the three ENSO indices (Niño-3 *I*_*N3*_, Niño-3,4 *I*_*N34*_ and Niño-4 *I*_*N4*_) over the period 1870 – 2022. It should be noted that the first half of the data is less accurate due to more sparse observations, while the data set after approximately 1980 gets more accurate due to the start of the satellite measurements. Therefore, we perform the same analysis of the intervals 1870–1945 and 1946–2022 separately, in order to check whether the spectral causality quantifiers are persistent through time with the results shown in SI. The AMO index is denoted here as $x_{1} = I_{A}$ and an ENSO index as $x_{2}$ = *I*_*N3*_, *I*_*N34*_ or *I*_*N4*_.

**Fig. 3:**
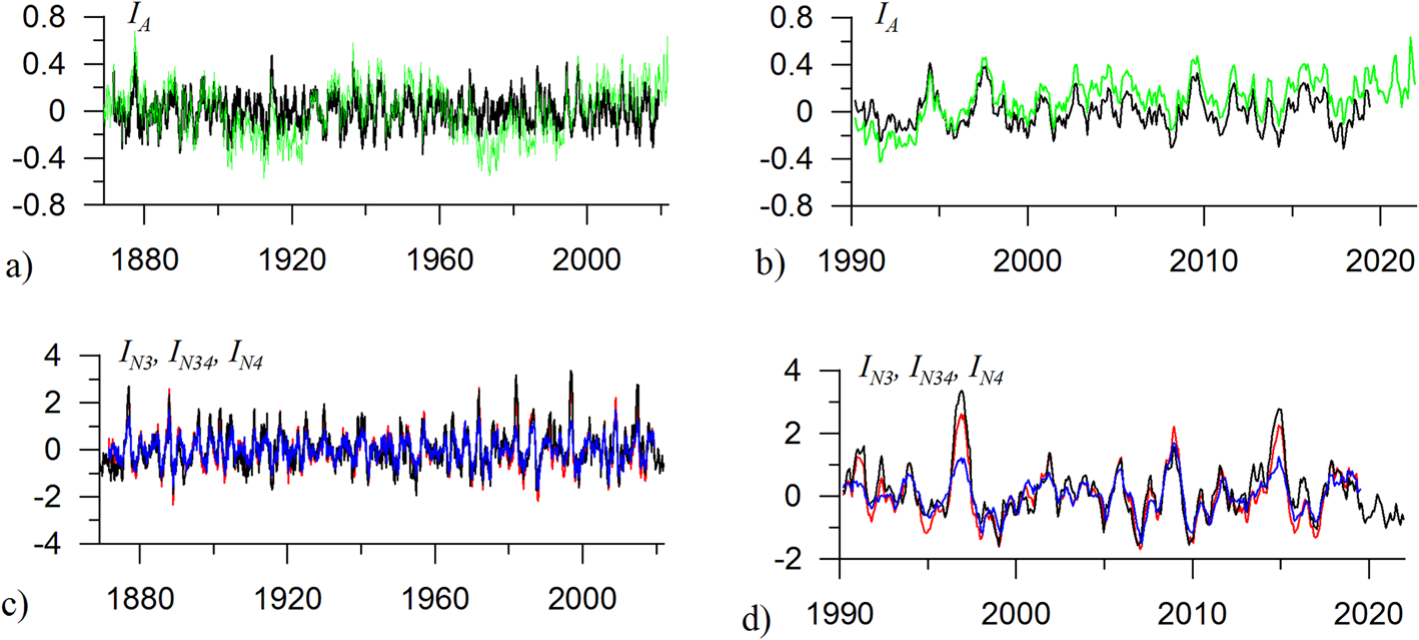
Time series of the indices AMO (**a**, **b**) and El-Niño (**c**, **d**): (**a**, **b**) the original index AMO (thin green lines) and that index after the subtraction of the 5-year moving average (black lines); (**c**, **d**) the indices Niño-3 (black lines), Niño-3,4 (red) and Niño-4 (blue), all three indices after subtraction of the 5-year moving average. Left panels (**a**, **c**) present full time series, right panels (**b**, **d**) – their magnified short segments.

**Fig. 4:**
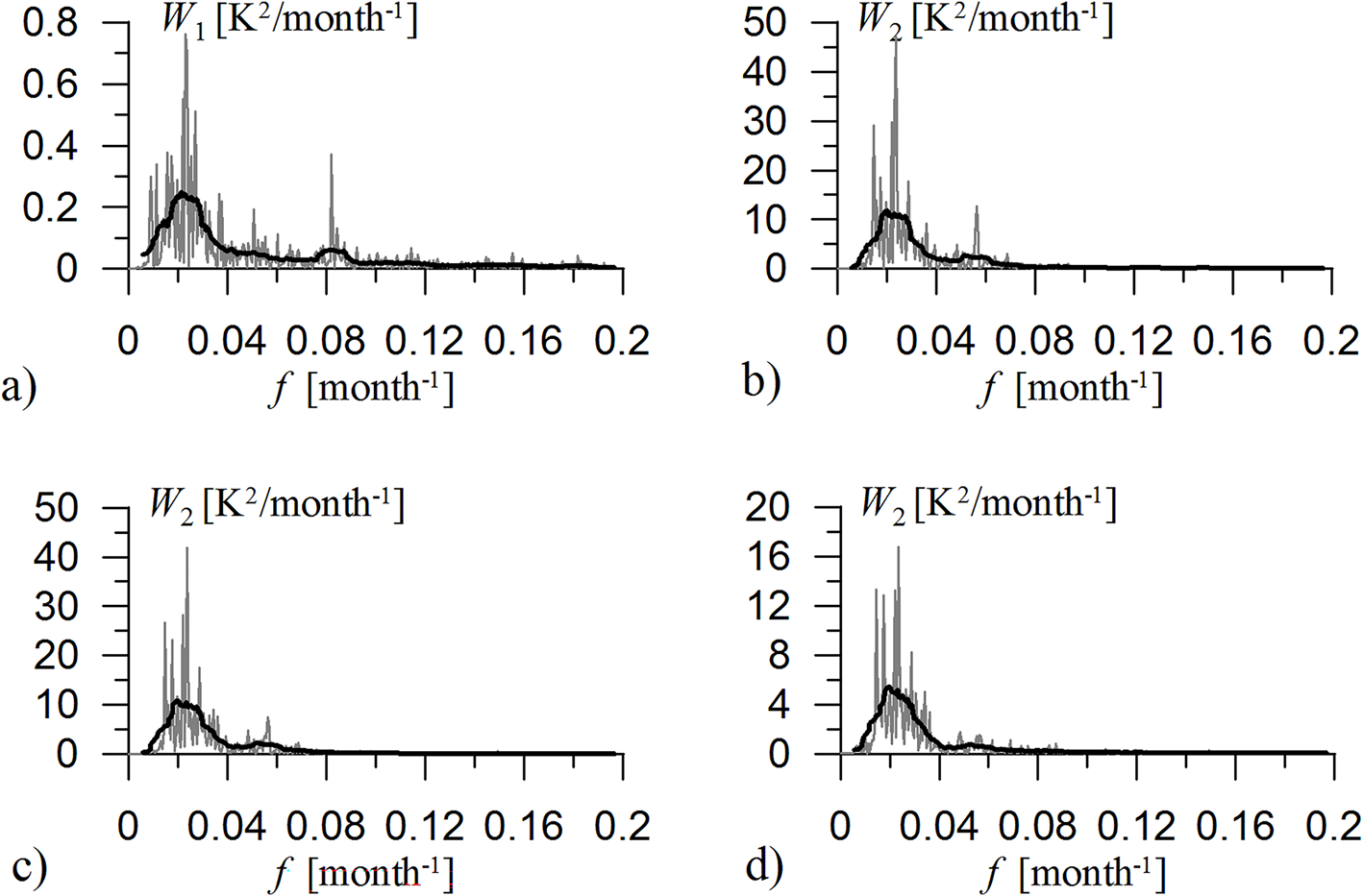
Estimates of the power spectral density for the “fast” components (after subtraction of the 5-year moving average) of the indices AMO (**a**) and El-Niño (**b** – Niño-3, **c** – Niño-3,4, **d** – Niño-4). Grey lines show periodograms, black lines – periodograms smoothed with rectangular window of the length 0.01 month^-1^.

To study the fast (intradecadal) variability, we have subtracted the *M*-year moving average from the original signals *I*_*A*_ and *I*_*N3*_, *I*_*N34*_, *I*_*N4*_. The basic choice is *M* = 5 years which efficiently damps oscillations with periods considerably greater than 5 years. The amplitude characteristic of such filter is not very steep, so the spectral components with periods moderately exceeding 5 years decrease moderately (roughly, the components with periods of up to 7–8 years pass the filter, see SI, Sec. S1.2, Figs. S2–S4). Figure 3,a-d presents the time series under the analysis and Fig. 4,a-d shows their power spectral densities (periodograms are shown with grey lines, smoothed periodograms with black ones). In SI (Sec.S2.5), we present the time series and results of their analysis for *M* ranging from 3 to 10 years.

*Methods.* Different coupling quantifiers (DCEs^49–51^) for the two processes under study, i.e. DCEs in both directions ${{1} \to {2}}$ and ${{2} \to {1}}$, are estimated here via construction of empirical autoregressive (AR) models from the observed data over the interval 1870 – 2022. Short-term causal effects can be quantified via the Granger causality measure^52^ which is one-step-ahead prediction improvement in terms of mean-squared prediction error. Within the DCE viewpoint, that effect is formulated as a change of the one-step-ahead conditional variance of one process under a certain variation of the initial state of the other process, e.g..^12,49^ Since statistical significance of its difference from zero and, hence, of the directional coupling presence is estimated in a standard way, the Granger causality measure is estimated here to detect directional couplings with confidence. Among long-term causality measures, the spectral ones are here in the center of attention. The spectral causality measures in general include Granger – Geweke causality spectrum,^32–35^ directed transfer function,^36,37^ partial directed coherence^38,39^ and their multiple versions (e.g.^40,41^). Uncertainties and controversies related to the results of their application and interpretation are reflected in a hot discussion.^42–47^ The work^48^ resolves those controversies by showing that those spectral characteristics are just different spectral causal effects within the DCE framework. Here, the spectral causal effect of switching a coupling on/off is evaluated as the basic measure. To complement the study, the long-term causal effect (of switching a coupling on/off) on variance is also computed. The formulas used to find all the above directional coupling measures are presented below.

*Fitting AR models and estimation of short-term DCEs.* Short-term DCEs between the two processes characterized by time series ${\left\{ {x_{i} (t)} \right\},\;t = {1,}\;{2,}...,N,\;i = {1,2}}$, where *N* is the time series length, are determined as follows, e.g.,.^11,12^ We estimate how strongly the future of $x_{i}$ depends on the past of $x_{j}$ given the past of $x_{i}$, i.e. we find the relative prediction improvement in terms of mean squared error. For that, we first construct univariate (individual) AR models

2 $${x_{i} (t) = A_{i,0} + \sum\limits_{k = 1}^{{d_{i} }} {A_{i,k} x_{i} (t - k)} + \xi_{i} (t),\;i = 1,2}.$$

Denote empirical variance of the residual errors ${\xi_{i} }$ as ${\hat{\sigma }_{i}^{{2}} }$. Model coefficients are estimated with the least-squared technique. To determine the values of the dimension (order) ${d_{i} }$, we minimize the Schwarz information criterion $I_{i} = \frac{{\left( {N - d_{\max } } \right)}}{2}\ln \hat{\sigma }_{i}^{2} + \frac{{d_{i} + 1}}{2}\ln \left( {N - d_{\max } } \right)$^71^ by varying ${d_{i} }$ from 0 to ${d_{\max } }$, the choice of ${d_{\max } }$ is described below. Using the obtained ${d_{i} }$, we construct a bivariate (joint) AR model (1) with ${d_{j \to i} }$ being the dimension of the coupling component (i.e. the number of previous values of the other variable ${x_{j} }$) and ${\eta_{i} }$ being the residual model errors with the empirical variance ${\hat{\sigma }_{i|j}^{{2}} }$. The relative prediction improvement ${G_{j \to i} = {{(\hat{\sigma }_{i}^{{2}} - \hat{\sigma }_{i|j}^{{2}} )} \mathord{\left/ {\vphantom {{(\hat{\sigma }_{i}^{{2}} - \hat{\sigma }_{i|j}^{{2}} )} {\hat{\sigma }_{i}^{{2}} }}} \right. \kern-0pt} {\hat{\sigma }_{i}^{{2}} }}}$ is a short-term (one-step) quantifier of the coupling ${j \to i}$ which represents a one-step response of ${x_{i} }$ to the variation of the initial state of ${x_{j} }$. We use the Fisher *F*-test with ${\left( {N - d{}_{\max },d_{j \to i} } \right)}$ degrees of freedom to estimate the statistical significance level *p* of the inferred nonzero value of ${G_{j \to i} }$ for each concrete ${d_{j \to i} }$. To select ${d_{j \to i} }$, we minimize the quantity ${d_{j \to i} p}$ while varying ${d_{j \to i} }$ from 0 to ${d_{\max } }$. If its achieved minimal value ${p_{\min } }$ is less than a small threshold (here 0.05), one infers a nonzero prediction improvement and hence the coupling ${j \to i}$ at the level of ${p_{\min } }$ which implements the Bonferroni correction for multiple testing. The trial values of ${d_{i} }$ and ${d_{j \to i} }$ are varied in such interval that the number of estimated coefficients in the corresponding AR model remains much less than the time series length, ${d_{\max } = 13}$ is chosen here. However, the coupling estimation results are quite robust to the variations of ${d_{\max } }$ up to the much larger value of 36 as shown in SI (Sec.S2.1 and Table S3).

For sufficiently large ${d_{i} }$ and ${d_{j \to i} }$ , a linear stationary AR model (1) with the least mean-squared error must provide delta-correlated (and desirably Gaussian) residual errors in order to be regarded adequate. This condition is checked here for all optimal AR models via estimation of the empirical autocorrelation functions of the residuals. If the properties of the residual errors differed from this condition, then a nonlinear (if the residual time series look stationary) or a nonstationary (if the residual time series exhibit, e.g., periodic non-stationarity or slowly rising trends) AR model might be necessary. Even if the residual errors meet the requirement of delta-correlatedness and look stationary, nonlinear and non-stationary models may still be fitted to check whether they can better describe finer details of the data. However, a search for a model within such wider classes of models may well make the inverse problem at hand ill-posed^72^. Thus, such a step often deserves a close attention but requires much effort, so it is necessary to be performed at once only if the linear stationary AR model is obviously non-adequate.

Autocorrelations of the time series under study do not strongly affect the estimates of statistical significance, since the autocorrelation times are much less than the time series length so the latter include many characteristic periods of both processes. Since the residual errors in both processes appear delta-correlated (see “Results”), the number of degrees of freedom on the *F*-test may be left ${\left( {N - d{}_{\max },d_{j \to i} } \right)}$ which is standard for the case of independent observations in regression estimation, without any correction for autocorrelations in the predictor variables.

Robustness of all estimation results is checked here by variations of model dimensions (selecting several values other than the optimal ones), filter cut-off frequency (selecting different moving window lengths *M*), part of time series used for model fitting (the entire observation period, its first and second halves), and some other parameters as presented in SI (Sec. S2, S4).

*Estimation of the long-term causal effects.* The basic causality quantifier used here is the spectral effect of the coupling $j \to i$^48,50,73^ which is found for both directions $2 \to 1$ and $1 \to 2$ and denoted $S_{j \to i} (f)$ where *f* is the frequency in units of month^-1^. This effect is the relative difference between the power spectral density $W_{i} (f)$ of the process ${x_{i} }$ for the coupling coefficients $b_{i,k}$ equal to their empirical estimates and equal to zero (under other equal conditions including the estimated values of the opposite coupling coefficients $b_{j,k}$):

3 $${S_{j \to i} (f) = \frac{{W_{i} (f) - W_{{i\left| {b_{i,k} = 0} \right.}} (f)}}{{W_{{i\left| {b_{i,k} = 0} \right.}} (f)}}}.$$

In Eq. (3), additional conditions under which the power spectral density $W_{i} (f)$ is determined are shown after the vertical line in the subscript. In other words, the spectral causal effect ${j \to i}$ is defined as the change of the power spectral density of the process $x_{i}$ under “switching” the coupling ${j \to i}$ “on/off” in the AR model. Switching the coupling ${j \to i}$ off is implemented via zeroing the coefficients $b_{i,k}$ (for all *k* > 0). $W_{1} (f)$ and $W_{2} (f)$ in Eq. (3) are computed via multiplying both sides of Eq. (1) by the complex exponential function $e^{{{\mathrm{i2}}\pi fn}}$, summing over all integer *n* to get Fourier transforms, multiplying both sides by their transpose, and taking expectation of both sides to get linear algebraic equations for the two power spectra and the cross-spectrum (e.g.^36^). Thereby one express $W_{1} (f)$ and $W_{2} (f)$ just via the AR coefficients ${a_{i,k} ,b_{i,k} ,a_{j,k} ,b_{j,k} }$. Then, ${W_{{1\left| {b_{1,k} = 0} \right.}} (f)}$ and ${W_{{2\left| {b_{2,k} = 0} \right.}} (f)}$ are computed via the formulas for $W_{1} (f)$ and $W_{2} (f)$ where coupling coefficients are taken as ${b_{1,k} = 0}$ and ${b_{2,k} = 0}$, respectively.

Along with the full spectral causal effect (3), we have studied the change of the spectral peak sharpness^73^. Denote ${R_{i} = \frac{{\mathop {\max }\limits_{f} W_{i} (f) - W_{i} (0)}}{{W_{i} (0)}}}$ the relative difference between the spectral peak of $x_{i}$ and the power spectral density at zero frequency. The AR models obtained here do not exhibit more than one spectral peak at a nonzero frequency, so $W_{i} (0)$ is the spectral minimum closest to the spectral peak and can be considered as a “noise floor”. If there is no peak at a non-zero frequency, we define ${R_{i} = 0}$. For example, ${R_{i} = 1}$ corresponds to the situation where the spectral peak is twice as high as (i.e. exceeds by 100%) the power spectral density at zero frequency. A spectral peak is called “sharp” here, if ${R_{i} \ge 0.5}$. Otherwise, it is “blurred”. Then, the dynamical causal effect with respect to the spectral peak sharpness is defined as

4 $${R_{j \to i} = R_{i} - R_{{i\left| {b_{i,k} = 0} \right.}} }.$$

We also estimate the spectral effect of a unidirectional coupling $S^{\prime}_{j \to i} (f)$, i.e. the effect $S_{j \to i}$ under an additional condition of zero opposite coupling $i \to j$ which coincides with the Granger – Geweke spectrum for the case of two processes as shown in^48^. It reads

5 $${S^{\prime}_{j \to i} (f) = \frac{{W_{{i\left| {b_{j,k} = 0} \right.}} (f) - W_{{i\left| {b_{i,k} = 0,b_{j,k} = 0} \right.}} (f)}}{{W_{{i\left| {b_{i,k} = 0,b_{j,k} = 0} \right.}} (f)}}}.$$

For a fuller analysis and comparison with the previous results^11^, we compute also the change in the stationary variance ${{\mathrm{var}} [x_{i} ]}$ (integral power of the fluctuations of the variable $x_{i}$ over a time interval much longer than characteristic time scales of the process under consideration) under switching the coupling ${j \to i}$ on/off. The DCE ${j \to i}$ with respect to the stationary variance is defined as the relative difference between the values of the variance

6 $${C_{j \to i} = \frac{{{\mathrm{var}} [x_{i} ] - {\mathrm{var}} [x_{i} \left| {b_{i,k} = 0} \right.]}}{{{\mathrm{var}} [x_{i} \left| {b_{i,k} = 0} \right.]}}}.$$

This coupling characteristic is similar to that used in^74^.

## Supplementary Information

Supplementary Information 1.

## Data Availability

AMO index I_A is available at [https://www.psl.noaa.gov/data/timeseries/AMO/] AMO index I_{A2}: https://www1.ncdc.noaa.gov/pub/data/cmb/ersst/v5/index/ersst.v5.amo.dat ENSO indices (HadISST data): https://psl.noaa.gov/data/timeseries/month/
