# Supplementary material for: Contribution of North Atlantic temperature variability to El Niño cyclicity as revealed by spectral causality estimates
Source: Sci Rep. 2026 Apr 20;16:18231. doi: 10.1038/s41598-026-48274-z (PMC13261045; doi:10.1038/s41598-026-48274-z)
Supplement: Supplementary file 1 — Supplementary Information 1. [file 41598_2026_48274_MOESM1_ESM.zip › 41598_2026_48274_MOESM1_ESM/SupplInf_SciRep_revision2.pdf]

Supplementary information to "Contribution of North Atlantic temperature variability to El Niño cyclicity as revealed with spectral causality estimates"

I. I. Mokhov[1,2] and D. A. Smirnov[1,3]

[1] Obukhov Institute of Atmospheric Physics of the Russian Academy of Sciences, 3 Pyzhevsky Per., Moscow 603950, Russia; mokhov@ifaran.ru

[2] Department of Physics, Lomonosov Moscow State University, Leninskiye Gory, Moscow 119991, Russia

[3] Saratov Branch of Kotelnikov Institute of Radioengineering and Electronics of the Russian Academy of Sciences, 38 Zelyonaya St., Saratov 410019, Russia; smirnovda@yandex.ru

This supplementary material presents the description of the data used and their characteristics including details of the model fitting procedure (Sec. S1), evaluation of robustness of the results to variations of different parameters of the methods (Sec. S2), details of the coupling estimation results for the three different ENSO indices (Sec.S3), and comparison of the coupling estimation results for different time epochs (Sec.S4).

S1. Data and their characteristics

S1.1. Residual annual cycle. Both AMO indices I_A and I_{A2} under study contain weak annual cycle (12-month component) as revealed by the average value of the index for each calendar month over the entire period 1870 – 2022 (thin grey line in Fig.S1). Seemingly, this is because the long-term mean over a 30-year reference period (rather than over the entire 143-year period) was subtracted from the original SST data. In this work, the remaining 12-month component is removed before the analysis. The removal of the annual cycle weakly changes the signal: the time series for the index I_A before and after the removal are shown in Fig.S1 (thick black and dashed blue lines, respectively). The power spectrum changes weakly too: despite the removal of the component at the frequency of 0.083 month^{-1} (i.e. at the period of 12 months), the component at the close frequency 0.082 month^{-1} remains (see Fig.2 for the power spectrum of the index I_A).

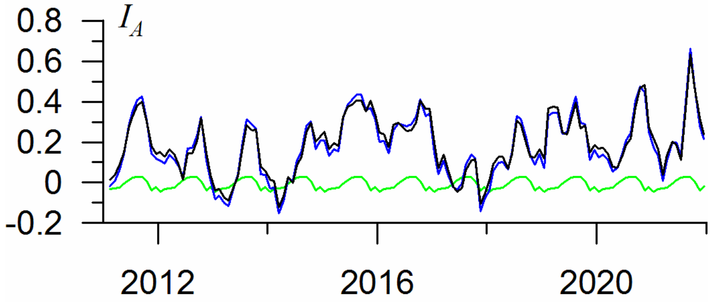

Fig. S1. A segment of the time series for the AMO index I_A before (black line) and after (blue line) the removal of the residual annual cycle (green line).

Removal of the residual annual cycle from the AMO index weakly influences the results of the AR model fitting and the DCE estimation (see Section S2.4). Therefore, all results are shown here in

detail for the index I_A after the removal of the residual annual cycle (except for Section S5). This is very similar for the AMO index I_{A2} (see Section S2.5).

As for the ENSO indices, they do not contain any discernible residual annual cycle.

S1.2. Different high-pass filters. Estimates of the power spectral density for the time series of the indices AMO I_A and El-Niño I_{N34} are shown in Figs.S2 and S3, respectively. In each of these figures, the panel (a) shows the periodogram of the original signal, i.e. before high-pass filtering. The panels from (b) to (g) in Figs.S2 and S3 present periodograms for rising cut-off frequency, i.e. for a decreasing value of M which is the window length for computing the moving average. It is straightforward to obtain that the high-pass filter with such rectangular window does not change the phase of the Fourier component at each frequency f and multiplies its amplitude by the factor of $1-\sin(\pi fM)/(\pi fM)$ (see Fig.S4 for the amplitude-frequency and amplitude-period characteristics of the filter with M = 5 years). Thus, the power at the period of 2M (and greater) decreases down to 13 % of its original value (and stronger), i.e. it is basically filtered out, while the power at the period of 1.5M (and 1.3M) decreases only down to 34 % (to 53 %), i.e. it passes the filter to a significant extent. So, one can roughly say that the filter retains the periods of 1.3M – 1.5M and shorter.

The periodogram of the unfiltered AMO index I_A (Fig.S2,a) exhibits peaks at the periods of approximately 77 years (the main peak), 9 years (the second peak) and 43 months (the third peak, the frequency of 0.023 month^{-1}). The high-pass filtering with M = 10 years retains only two peaks, and the peak at the period of 43 months is much lower. For the window lengths of M = 7 years and M = 6 years, both peaks are of approximately the same height. For the window length of M = 5 years, the peak at the period of 9 years gets much lower and more blurred, while the 3-4-year periodicity remains weakly decreased by the filter. Further decrease of the filter window length M decreases low-frequency components even stronger, including already the 3-4-year periodicity. Thus, the peak at the period of 43 months for the window length of M = 4 years is close in its height (and in its ratio to the peak at the period of about 1 year) to the same peak for the window length of M = 5 years. For the window length of M = 3 years, the 3-4-year periodicity is already strongly decreased.

Figure S2,h presents smoothed periodograms of the AMO index for all the filter windows. Such smoothed power spectral density estimate for the window length of M = 5 years (green) is close to the estimate for M = 6 years (cyan), while the estimate for the window length of M = 4 years (yellow) differs much stronger (with respect to the power in the interval of periods of about 3-4 years). This analysis prompts us that it is most adequate to use the filter window length of M = 5 years to study the NATV in the interval of periods of about 3-4 years (and less).

Figure S3 shows analogous power spectra estimates for the ENSO index I_{N34}. In particular, the periodogram of the unfiltered signal exhibits three peaks at the periods of 68 months, 44 months and 35 months. They are maintained after filtering with the window length of M = 6 years. The window length of M = 5 years leads to some decrease of the first peak, so the second of them start to dominate. A further decrease of the filter window length decreases the low-frequency components. Smoothed periodograms (Fig.S3,h) show that the power spectra are close for the filtered signals with the filter window lengths of M = 7 years (blue) and M = 6 years (cyan). The window length of M = 5 years gives the power spectrum estimate (green) which is somewhat different for the periods exceeding 50 months, but overall quite similar. The window length of M = 4 years (yellow) leads already to

a considerable decrease of variability for the period of about 40 months and, therefore, to a considerably different power spectrum. Thus, the window length of M = 5 years seems to be adequate for the analysis of the ENSO index I_{N34} in the range of periods of 3-4 years (and less). This value is used in this work as the basic choice to filter both the AMO and the ENSO indices.

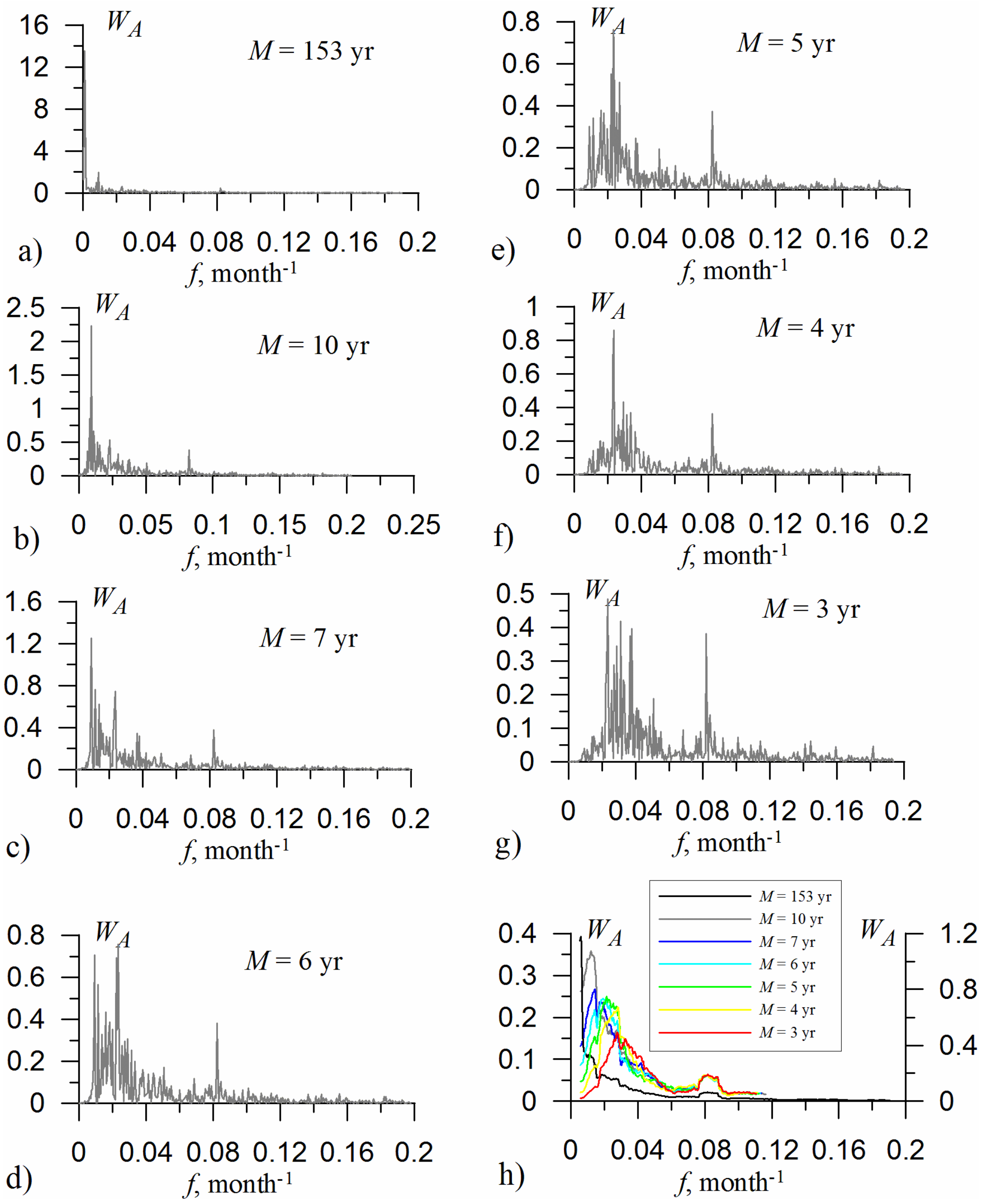

Fig. S2. Periodograms for the index I_A calculated from the time series over the interval 1870-2022 after high-pass filtering with different window lengths M (a-g). The panel (h) shows the smoothed periodograms, the right ordinate axis refers to the black line corresponding to the unfiltered signal. Smoothing in the frequency domain is done with the rectangular window of the width of 0.01 month^{-1} which covers 21 subsequent frequency components of the periodogram (i.e. of the discrete Fourier transform) for the frequency resolution determined by the time series length of N = 1836 months.

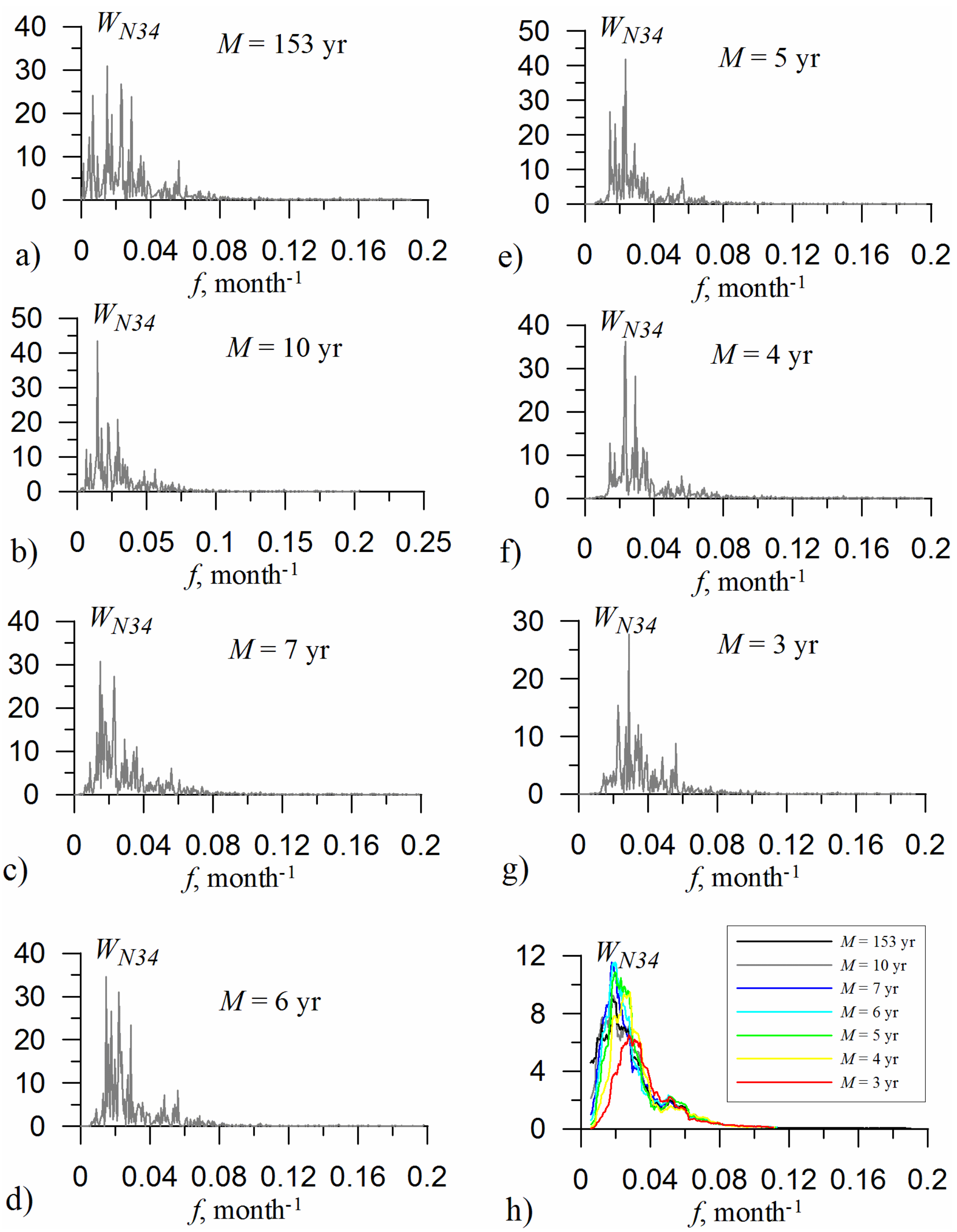

Fig. S3. Periodograms for the index I_{N34} high-pass filtered with different window lengths M (a-g). The panel (h) shows the smoothed periodograms. Notations are the same as in Fig. S2.

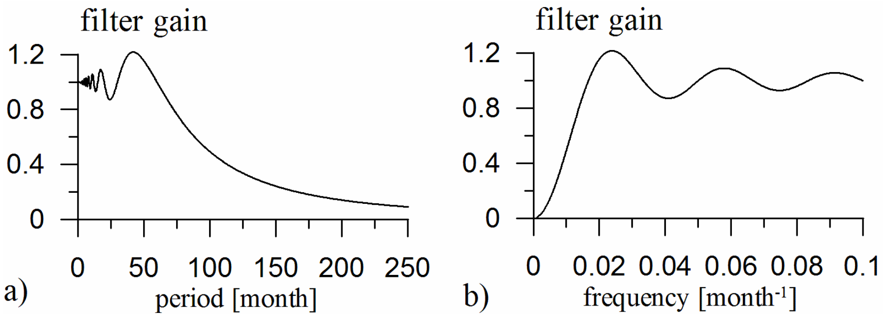

Fig. S4. Amplitude gain factor of the high-pass moving average filter with the moving window length M = 60 months versus period (a) or frequency (b) of a Fourier component.

Figure S5 shows the time series of the AMO indices I_A and I_{A2} before and after high-pass filtering with the window length of $M = 5$ years. Figure S5,a shows the index I_A over a short interval where both versions slightly differ. Figures S5,b shows indices I_A and I_{A2} after high-pass filtering over the same short interval where they slightly differ from each other too. Figure S5,c shows these two indices over a long interval where they differ considerably. It means that they differ from each other with respect to their long-period components. Over a short interval (Fig.S5,d), this is manifested roughly as their shift along the ordinate axis with respect to each other. Thus, one should expect similar results of the coupling analysis under the usage of any of these two indices with the filter window length of $M = 5$ years. This is confirmed in this work (see Section S2.4).

Figure S6 analogously illustrates the time series of the ENSO indices. The high-pass filtering with the window length of $M = 5$ years slightly changes the signal properties over a short interval (Fig.S6,a). The three ENSO indices do not strongly differ from each other, though some differences (especially for the index I_{N4}) are present (Fig.S6,b).

Figure S7 shows the cross-correlation function between the high-pass filtered indices I_A and I_{N34}. The cross-correlations are significant, their maximum corresponds to the ENSO index leading with the time shift of 6 months. A similar conclusion follows from the estimates of the cross-spectrum and Fourier coherence function (Fig. S8) which exhibit a peak at the period of about 3-4 years (Fig.S8,a) with the phase difference of about 1 radian (Fig.S8,b). The ENSO signal leads with the time shift of about 1/6 of the basic period, the latter equals about 6 months for the spectral peak at 37-38 months which agrees with the cross-correlation peak at the time lag of 6 months.

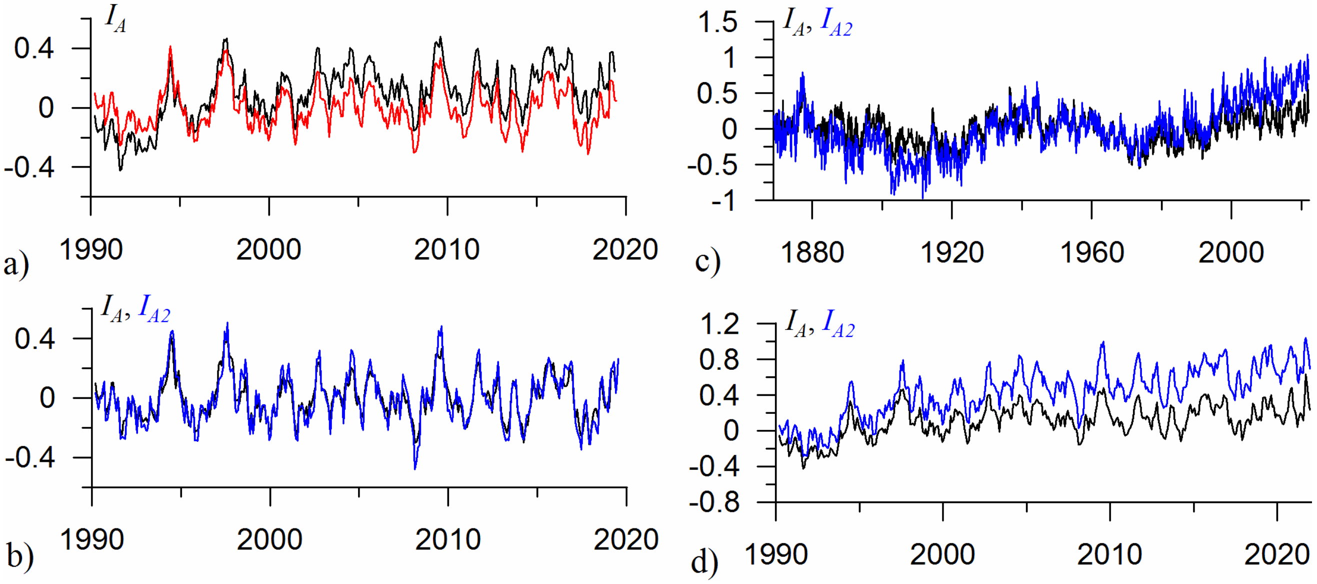

Fig. S5. Time series of the AMO indices: a) the unfiltered index I_A (black) and high-pass filtered index I_A with the window length of $M = 5$ years (red); b) high-pass filtered signals I_A (black) and I_{A2} (blue); c) the unfiltered signals I_A (black) and I_{A2} (blue) over the entire period 1870 – 2022; d) the unfiltered signals I_A (black) and I_{A2} (blue) over the period 1990 – 2022.

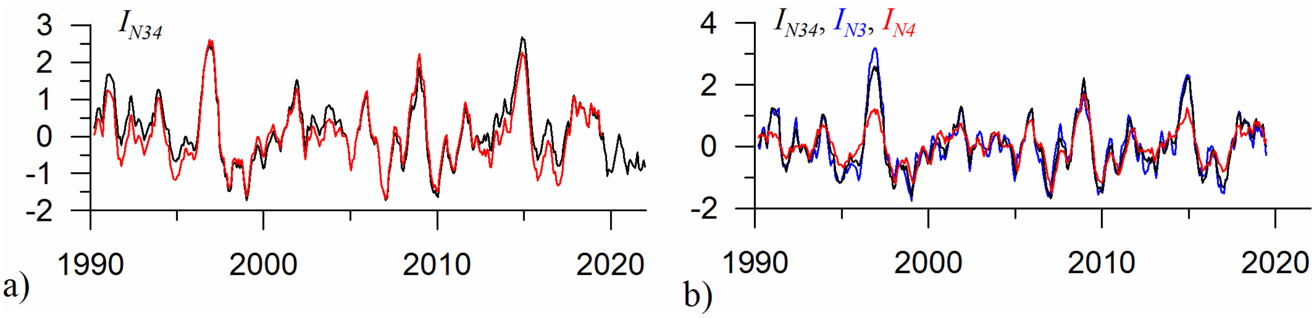

Fig. S6. Changes of the El-Niño indices over the interval 1990–2022: a) the unfiltered index I_{N34} (black) and the same signal high-pass filtered with the window length of M = 5 years (red); b) the high-pass filtered signals I_{N34} (black), I_{N3} (blue) and I_{N4} (red).

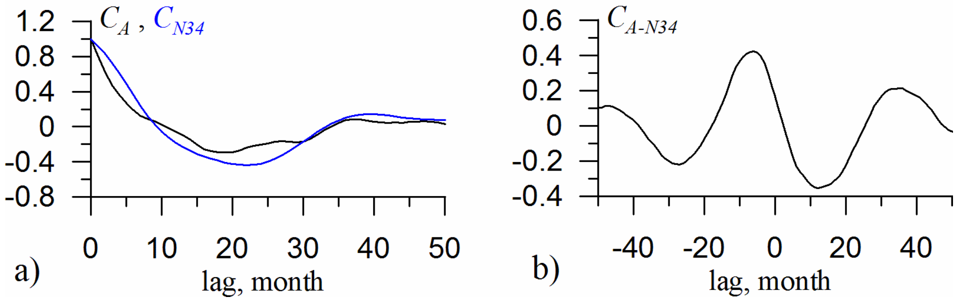

Fig. S7. Autocorrelation functions of the high-pass filtered signals I_A and I_{N34} (black and blue, respectively) (a) and their cross-correlation function (b).

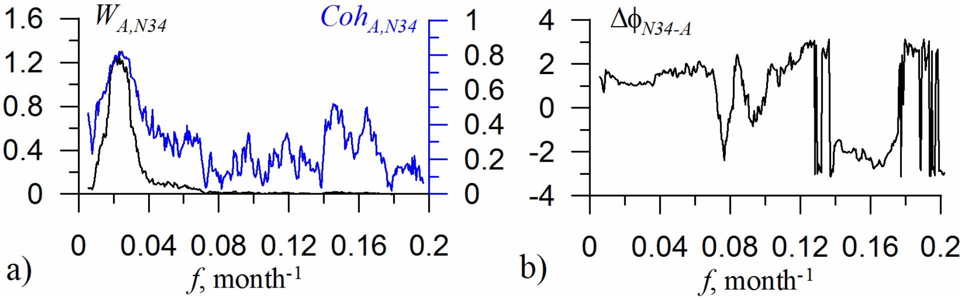

Fig. S8. Cross-spectral characteristics of the high-pass filtered signals I_A and I_{N34}: (a) cross-periodogram (black) and coherence function (blue); (b) the phase spectrum (phase difference between the two signals equal to the phase of the cross-periodogram).

S1.3. Empirical models. Optimal AR models (1) for different ENSO indices are obtained for the dimensions shown in Table S1. Figure S9 shows details of the model selection procedure for the indices I_A and I_{N34}: the Schwarz information criterion (Fig.S9,a,b) and statistical significance level (Fig.S9,c,d) versus the dimensions of the individual and coupling components, respectively. Optimal model dimensions are selected as the minimum points. Figure S9,c,d shows that the Granger causality estimated as the relative prediction improvements in both directions are significantly non-zero at significance levels less than 0.00001. Table S1 present the values of those prediction improvements. For the coupling ENSO → NATV and any ENSO index, the one-month-ahead prediction error is $G_{2\to 1} \approx 4\,\%$. According to the point estimates in Table S1, this coupling is stronger for the index of the eastern equatorial Pacific region Niño-3, somewhat weaker for the region Niño-3,4 and even weaker for the Niño-4, though the differences are small and statistically insignificant. For the opposite direction NATV → ENSO, the largest prediction improvement $G_{1\to 2} = 3.2\,\%$ is obtained for the

index Niño-3, considerably weaker $G_{1\to2} = 2.3\,\%$ for the index Niño-3,4 and even weaker $G_{1\to2} = 1.7\,\%$ for the index Niño-4.

Table S1. Dimensions (orders) of the optimal AR models and Granger causality estimates for the three ENSO indices for the period 1870 – 2022, $d_1 = 1, d_2 = 6$ for all models.

	I_{N3}	I_{N34}	I_{N4}
$d_{2\to1}$	5	3	3
$d_{1\to2}$	12	12	6
$G_{2\to1}$, %	4.4	4.3	4.1
$G_{1\to2}$, %	3.2	2.3	1.7

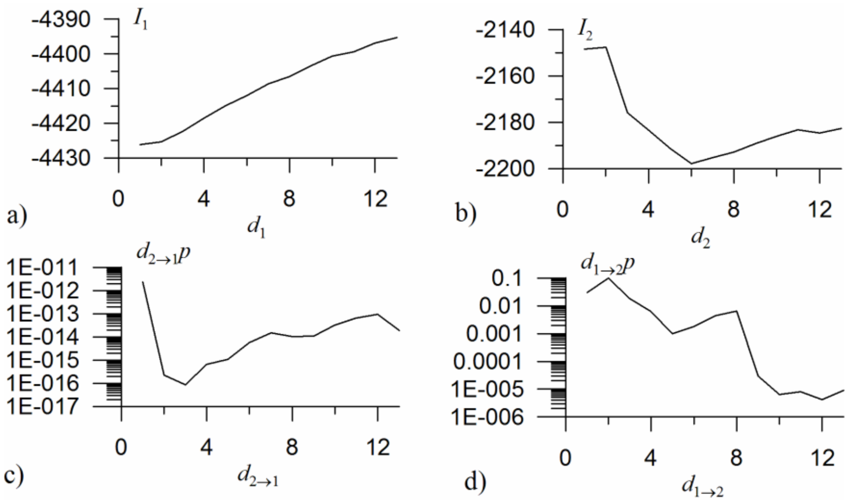

Fig. S9. Selection of an optimal empirical model for the indices I_A and I_{N34}: (a,b) Schwarz information criterion versus the dimension of the individual component; (c,d) Bonferroni-corrected significance level of the conclusion about the coupling presence versus the dimension of the coupling component.

Table S2 presents coefficients of the optimal AR model for the indices I_A and I_{N34} (achieved at $d_1 = 1, d_2 = 6, d_{2\to1} = 3, d_{1\to2} = 12$) with their standard deviations. Some of the coupling coefficients $b_{2,i}$ differ from zero significantly as it is evidenced by the Fisher F-test (Fig.S9,d). Figure S10 presents residual errors of the optimal AR model (Fig.S10,a,d) and their properties including autocorrelation functions (Fig.S10,b,e), empirical probability distribution functions (histograms, Fig.S10,c,f) and cross-correlation function (Fig.S10,g) which confirm that the properties of the residuals do not differ significantly from the properties of Gaussian white noise.

Table S2. Coefficient estimates for the AR model (1) with their standard deviations. The AR model is obtained from the time series of the indices I_A and I_{N34}.

I	$a_{1,i}$	$b_{1,i}$	$a_{2,i}$	$b_{2,i}$
1	0.77 ± 0.015	-0.010 ± 0.006	0.89 ± 0.02	-0.04 ± 0.09
2	–	0.017 ± 0.009	0.13 ± 0.03	0.06 ± 0.11
3	–	0.017 ± 0.007	-0.07 ± 0.03	-0.07 ± 0.11
4	–	–	0.003 ± 0.03	-0.004 ± 0.11
5	–	–	-0.02 ± 0.03	-0.15 ± 0.11
6	–	–	-0.09 ± 0.02	-0.13 ± 0.11
7	–	–	–	0.10 ± 0.11
8	–	–	–	0.17 ± 0.11
9	–	–	–	-0.17 ± 0.11
10	–	–	–	-0.12 ± 0.11
11	–	–	–	0.02 ± 0.11
12	–	–	–	-0.16 ± 0.08

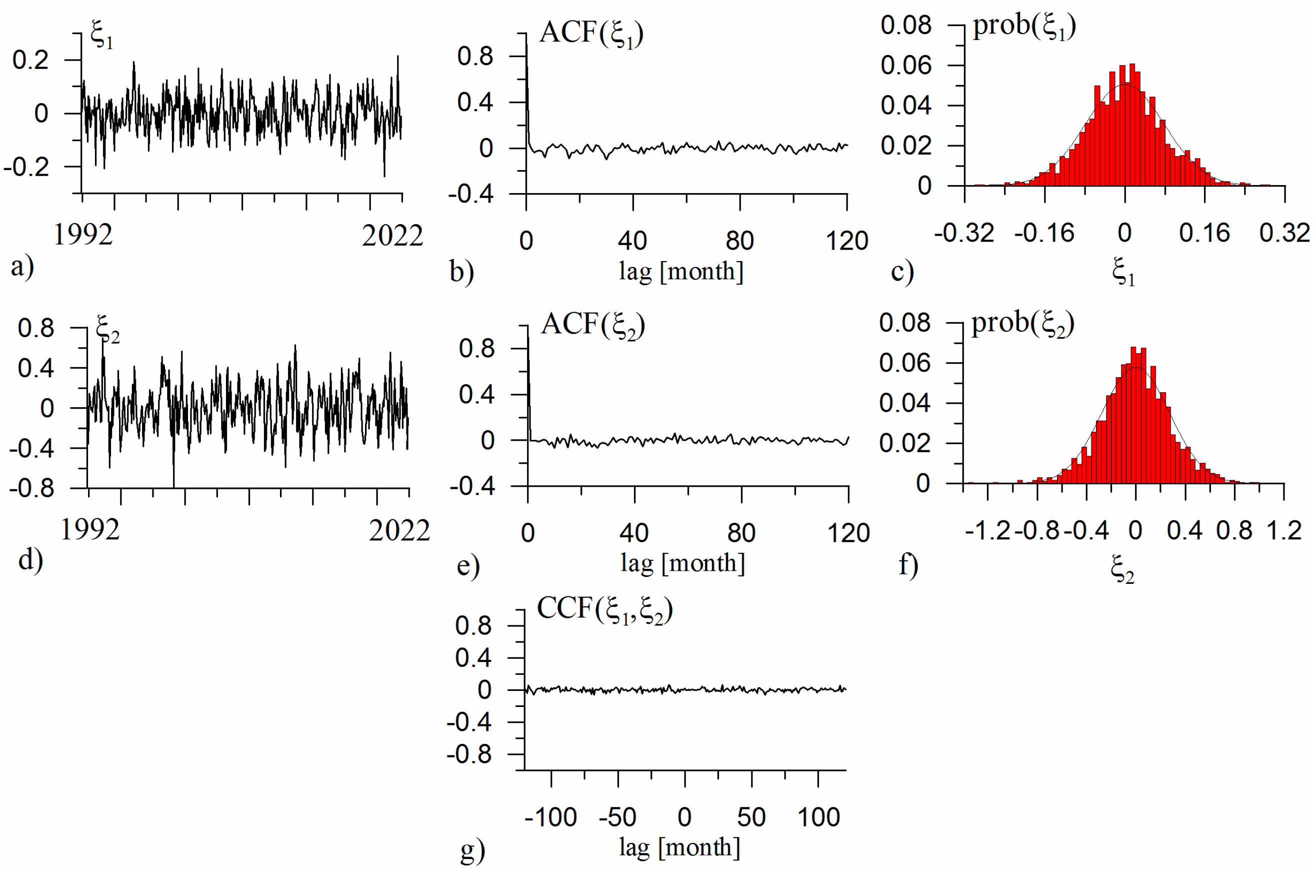

Fig. S10. Residual error analysis for the optimal AR model specified in Table S2: (a,d) 30-yr parts of the time series of the residual errors; (b,e) autocorrelation functions of the residuals; (c,f) empirical probability distribution functions (histograms) of the residuals (red) and fitted Gaussian curves (black); (g) cross-correlation function of the residuals.

S2. Robustness of the coupling estimation results

S2.1. Robustness to variations of the model dimensions. Can empirical AR models of different dimension (orders), apart from the optimal one, reproduce power spectral densities of the climate indices under study well enough? Do they give close estimates of spectral causal effects? Figure S11 presents the results for the AR models with the dimensions of the coupling component equal to $d_{1\to2} = 10$ (Fig. S11,a,b), $d_{1\to2} = 5$ (Fig. S11,c,d) and $d_{1\to2} = 1$ (Fig. S11,e,f) which correspond to local minima of the significance level shown in Fig. S8,d. It appears that the main result presented in the main text is quite robust: the spectral DCEs remain similar to the estimates for the optimal model with $d_{1\to2} = 12$ for the AR models with $d_{1\to2} = 10$ and even $d_{1\to2} = 5$ though the observed power spectra are reproduced by those models somewhat worse than by the model with $d_{1\to2} = 12$. However, $d_{1\to2} = 1$ appears to be too small, since the coupling in the AR model weakly influences the shape of the power spectral densities of the NATV and the ENSO, and even the full AR model with a bidirectional coupling does not exhibit clear peaks at periods of about 3-4 years. Thus, a reasonably large $d_{1\to2}$ (e.g. equal to 5), even considerably less than the optimal value, appears sufficient to provide qualitatively the same conclusion about a key role of the bidirectional coupling in maintaining the spectral peaks of the NATV and the ENSO at periods of 3-4 years.

Increase of the maximal range $d_{\max}$ of trial dimensions from the above value of 13 up to the value of 36 has also been checked. The main results are robust to this parameter as well (Table S3).

Table S3. Dimensions $d_{1\to2}$, Granger causalities and spectral causal effects for the optimal AR models at different range of dimensions $d_{\max}$ (for the ENSO index Niño-3,4 over the entire period 1870 – 2022). Optimal values of the other dimensions are always the same: $d_1 = 1, d_{2\to1} = 3, d_2 = 6$. Granger causality in the direction 2 $\to$ 1 is almost the same for all $d_{\max}$ and is not reported also.

$d_{\max}$	13	14	15	18	21	24	30	36
$d_{1\to2}$	12	14	15	16	16	16	16	16
$G_{1\to2}$, %	2.3	2.5	2.6	3.0	3.0	3.0	3.0	3.0
$d_{1\to2}p$	$4\cdot10^{-6}$	$4\cdot10^{-6}$	$2\cdot10^{-6}$	$3\cdot10^{-6}$	$3\cdot10^{-6}$	$4\cdot10^{-6}$	$4\cdot10^{-6}$	$4\cdot10^{-6}$
$S_{2\to1}^{\max}$	1.4	1.5	1.6	1.6	1.6	1.6	1.6	1.6
$S_{2\to1}^{\min}$	-0.3	-0.3	-0.3	-0.4	-0.4	-0.4	-0.4	-0.4
$R_{2\to1}$	1.3-0.0	1.6-0.0	1.8-0.0	2.0-0.0	2.0-0.0	2.0-0.0	2.0-0.0	2.1-0.0
$S_{1\to2}^{\max}$	1.1	1.2	1.3	1.5	1.5	1.5	1.5	1.5
$S_{1\to2}^{\min}$	-0.2	-0.2	-0.2	-0.2	-0.2	-0.2	-0.2	-0.2
$R_{1\to2}$	2.3-0.2	2.6-0.3	2.7-0.3	2.8-0.3	2.8-0.3	2.8-0.3	2.8-0.3	2.9-0.4
$C_{2\to1}$	0.24	0.24	0.24	0.23	0.23	0.23	0.23	0.24
$C_{1\to2}$	0.26	0.29	0.31	0.33	0.33	0.33	0.33	0.33

Table S3 exhibits moderate differences between the coupling estimation results for any $d_{\max} \geq 16$ (where the optimal value of $d_{1\to2} = 16$ is attained) and our basic case of $d_{\max} = 13$ (where the optimal value is $d_{1\to2} = 12$). Namely, the estimated spectral effects differ only by 25 % or less, all qualitative features of the spectral effects plotted versus frequency are the same.

Still, the value of $d_{\max} = 13$ used in this work seems to be more reliable than $d_{\max} \geq 16$ to avoid overfitting of the AR models. Indeed, the number of estimated coefficients in each line of the largest AR model (1), i.e. $P = 2d_{\max} + 1$, should be much less than the number of statistically independent data intervals N' within the time series under study (in practice one often uses the rough empiric rule of $P \leq \sqrt{N'}$). The length of the time series is ≈ 1800 data points. Autocorrelation functions of both ENSO and NATV indices fall down to statistically insignificant values for the lag of 6 months (Fig.S7), hence subsequent time intervals of 6 data points can be considered as mutually independent. So, $2d_{\max} + 1$ must be less than $\sqrt{1800/6} \approx 17$, i.e. it is desirable to take $d_{\max} \leq 8$. The value of $d_{\max} = 13$ is somewhat greater, but it gives a model which reproduces the observed spectral power more accurately than $d_{\max} \leq 10$, while $d_{\max} = 13$ and $d_{\max} \geq 16$ give the results closer to each other. Moreover, the value of $d_{\max} = 13$ is closer to the desirable $d_{\max} \leq 8$ than the value of $d_{\max} \geq 16$. It is especially important for analyses of shorter data, e.g. half the time series under study (Sec. S4). Therefore, we have selected $d_{\max} = 13$ here, but AR models obtained with slightly larger $d_{\max}$ (and, hence, slightly greater $d_{1\to2}$) might be used also. It would not change the main results of this work.

S2.2. Robustness to moderate variations of the time series length. Directional couplings (the short-term DCEs and the DCEs with respect to stationary variance) between the processes under study were estimated in [12] for the dame data over the period 1870–2013, i.e. the data length was shorter than the current time series length by about 6 % (9 years). The optimal model was found at $d_{1\to2} = 10$, i.e. the precise coupling dimension value is moderately sensitive to such small variations of the time series length. Here, we have performed the spectral DCE estimation from that shorter time series as well. It is obtained that the resulting spectral DCEs (not shown) are almost indistinguishable from the main results shown in Fig.2, i.e. the spectral DCE estimates and the main conclusion about the "spectral role" of the bidirectional coupling are robust to the moderate variations of the time series length.

We have also checked even smaller time series down 75 years (half the full time series at hand). However, it deserves a special consideration, since one can already compare the results for the first and second half of the period under study and think of the slow change of the couplings and their effects. Still, right here we can state that the power spectral densities and spectral DCEs look qualitatively the same as those in Figures 1 and 2 (i.e. for the full time series) with some moderate quantitative differences. Hence, the main conclusion about the importance of bidirectional coupling for the existence of the observed spectral peaks in the intradecadal (interannual) range is robust even to quite strong variations of the time series length which is quite a non-trivial check since the corresponding considerable decrease of the data amount (given quite moderate data amount in the full time series) could lead to much larger statistical errors.

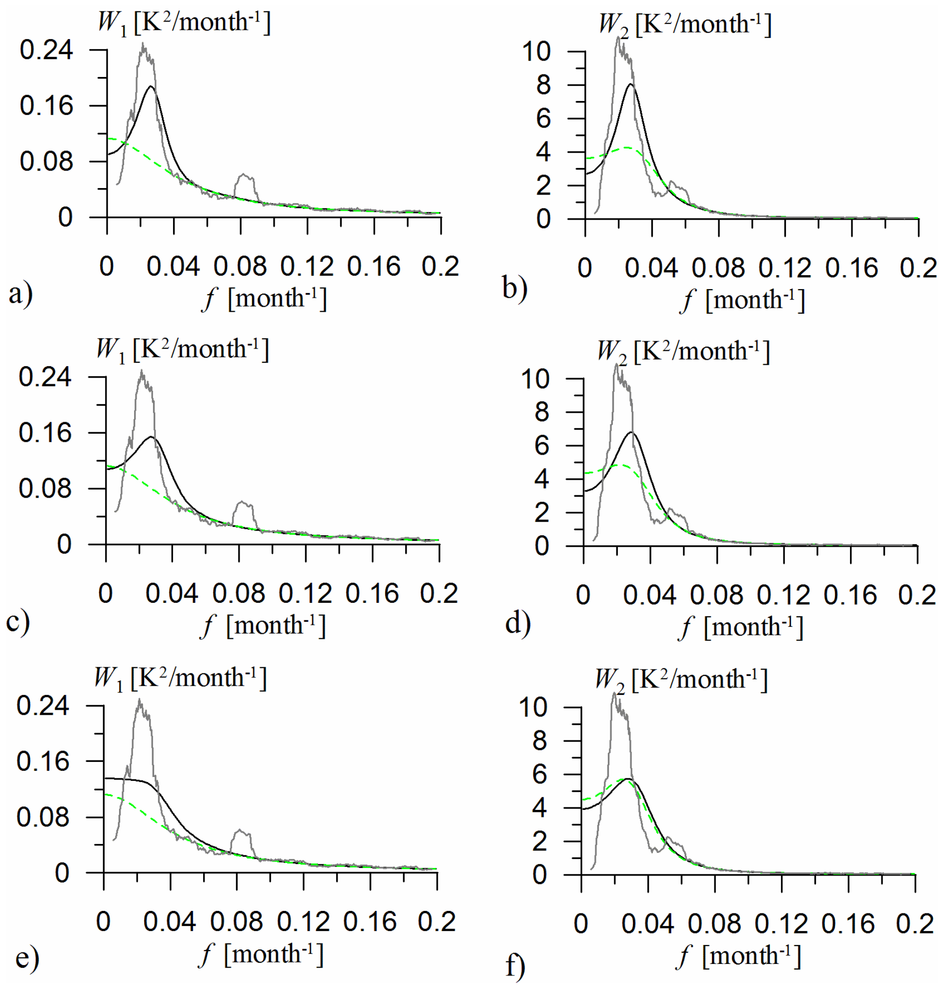

Fig. S11. Power spectral densities for the indices of the NATV I_A (a,c,e) and the ENSO I_{N34} (b,d,f) according to the full empirical AR model (black lines) and to the same AR model with zero couplings (green dashed lines) for different dimensions of the coupling component $d_{1\to2}$: (a,b) $d_{1\to2} = 10$; (c,d) $d_{1\to2} = 5$; (e,f) $d_{1\to2} = 1$. Grey lines show smoothed periodograms for the data under the analysis.

S2.3. Robustness to the filtering/non-filtering of the ENSO data. If the time series of an ENSO index is not high-pass filtered, then the long-period components are retained and the results exhibit certain differences. An optimal AR model is then achieved at $d_1 = 1$, $d_{2\to1} = 2$, $d_2 = 6$, $d_{1\to2} = 10$. The corresponding spectral DCEs are shown in Fig.S12 which evidences that the individual AR model for the ENSO does not have a spectral peak (even a blurred one) at any non-zero frequency. Switching the bidirectional coupling on leads to the arousal of the spectral peak for the model ENSO. Still, the quantitative difference of the spectral DCEs and the power spectra themselves from the case of high-pass filtered ENSO time series is not large (cf. Figs.1 and S12).

Since the full AR model reproduces the observed power spectral densities of the ENSO and the NATV signals somewhat worse than that for the high-pass filtered ENSO signal, the latter case (i.e. the same high-pass filtering of both the NATV and the ENSO signals) is chosen here as the basic one and all the results are shown for it.

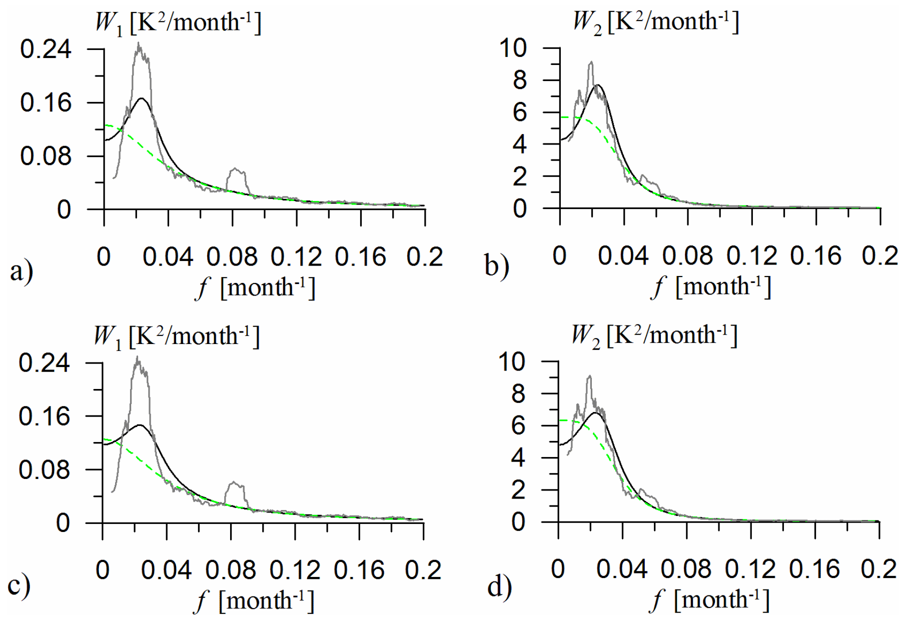

Fig. S12. Power spectral densities for the indices of the NATV I_A (a,c) and the ENSO I_{N34} (b,d) according the full AR model (black lines) and to the AR model without couplings (green dashed lines) estimated from the non-filtered ENSO data for different locally optimal dimensions: (a,b) $d_{1\to 2} = 10$; (c,d) $d_{1\to 2} = 5$. Grey lines show smoothed periodograms of the signals under study.

S2.4. Robustness to the removal/non-removal of the residual annual cycle from the time series of the AMO index. As it was mentioned above, the residual annual cycle was subtracted from the time series of the AMO index before the analysis. How strongly does this removal affect the results of the directional coupling estimation? Figure S13 presents the results of the AR modeling and the power spectral density estimation when the residual annual cycle is not subtracted from the time series of the AMO index under other equal conditions. These results are almost indistinguishable from the previous results shown in Fig.1 of the main text. Similarly, the spectral DCEs remain almost the same (not shown). So, the obtained spectral DCE estimates and the qualitative conclusion are robust to the removal/non-removal of the residual annual cycle from the AMO index.

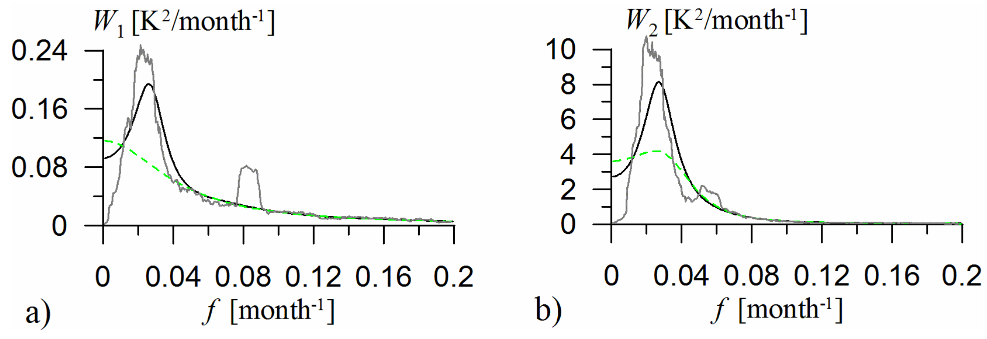

Fig. S13. Power spectral densities for the indices of AMO (a) and ENSO (b) according to the full AR model (black lines) and to the AR model without couplings (green dashed lines) estimated from the AMO index without subtraction of the residual annual cycle ($d_{1\to 2} = 10$). Grey lines show smoothed periodograms of the signals under study.

S2.5. Robustness to the variations of the window length M of the high-pass filter. To study robustness of the results to variations of the filter window length M (i.e. to the cut-off frequency), we have performed the same analysis for the time series high-pass filtered with different M ranging from 3 to 10 years. The results are shown in Figs. S14 and S15 and Table S3 which evidence that the spectral DCE estimates are moderately sensitive to this parameter of the method. The estimates for the window length of M = 4 years slightly differ from those for M = 5 years, while the difference is somewhat greater for M = 6 years. The windows of the lengths 3, 7 and 10 years give the coupling estimates considerably different quantitatively: prediction improvements are less significant (Table S4) and spectral DCEs are weaker (approximately twice), though the qualitative conclusion about the key role of the bidirectional coupling for the existence of intradecadal (interannual) spectral peaks for both processes remains. The differences are induced by the circumstance that the usage of the small M = 3 years considerably distorts the spectral components in the range of periods of the basic spectral peak (3-4 years) while the usage of the long window with M = 10 years retains too long-period spectral peaks (close to 10 years). If the peak under study in the range of periods of 3-4 years corresponds to real interacting oscillatory processes, then an adequate filter must remove superfluous (long-period) components and maintain necessary (belonging to the mentioned range of periods) components. It determines the obtained range of preferable filter window lengths from 4 to 5 years.

Table S4. Characteristics of the optimal AR models and the corresponding directional coupling estimates for the different filter window lengths *M*.

M, years	3	4	5	6	7	10
d_1	2	2	1	1	1	1
d_2	8	6	6	6	6	6
$d_{2\to1}$	3	3	3	2	2	2
$d_{1\to2}$	12	12	12	10	10	9
$G_{2\to1}$, %	3.8	4.5	4.3	3.4	3.0	2.6
$d_{2\to1}p$	$4\cdot10^{-15}$	$7\cdot10^{-17}$	$9\cdot10^{-16}$	$7\cdot10^{-14}$	$3\cdot10^{-12}$	$2\cdot10^{-10}$
$G_{1\to2}$, %	1.6	2.5	2.3	1.8	1.4	1.1
$d_{1\to2}p$	$8\cdot10^{-4}$	10^{-6}	$4\cdot10^{-6}$	$7\cdot10^{-5}$	0.001	0.008
$C_{2\to1}$, %	–	22.6	23.7	16.9	14.7	14.0
$C_{1\to2}$, %	–	26.8	26.1	17.6	13.7	9.3

The frequency range of the basic spectral peak is sufficiently robust characteristic: for different filter window lengths from 4 to 7 years, the spectral peak in smoothed periodograms of the observed signals covers the range of periods from 2.5 to 4 years, and the frequency of the spectral peak in the estimated AR models (1) is achieved at the period of 36-45 months for the NATV and 34-40 months for the El-Niño. Only the shortest window of 3 years strongly shifts the maximum to the boundary of this window: its period is approximately 30 months. Similarly, the longest window strongly affects the period of the spectral maximum. Hence, the spectral components under study seem to be intrinsic

to the processes under study (rather than being artificially imposed), and they are best separated with the high-pass filter with the moving window length of 5 years. Therefore, this is the basic choice for which all the results are presented in this work.

S2.6. Robustness to the variation of the AMO index. To study this point, we have performed the same analysis of directional couplings using the index I_{A2} according to the ERSSTv5 data over the same period 1870-2022 instead of I_A. An optimal model is achieved at $d_1 = 2$, $d_{2\to1} = 2$, $d_2 = 6$ and $d_{1\to2} = 12$. The DCE estimation results for this model and three models with smaller dimensions are presented in Fig. S16. The results for the best model with $d_{1\to2} = 12$ are quite close to the above results for the index I_A. Thus, the main conclusion is robust to the variation of the AMO index. However, the spectral DCE estimates are weaker already for a small decrease of $d_{1\to2}$, i.e. the results for the AMO index I_{A2} are somewhat less robust to the variations of the AR model dimensions.

We notice that the AR model constructed from the data for the AMO index I_{A2} reproduces the power spectrum of the ENSO somewhat worse than the model obtained from the data for the AMO index I_A. Therefore, the results for the AMO index I_A are presented in this work as the basic case.

S2.7. Robustness to statistical variations of time series (surrogate data tests). There is no direct way to test statistical significance of the obtained non-zero spectral causal effects along with the shape of their plots, including the concrete frequencies of their maxima. Still, as a rough substitute, we have performed surrogate data tests of two kinds. In any of them we generate 1000 pairs of independent time series, one of them as a substitute for the NATV index and the other one for the ENSO index. For each pair we perform the same coupling estimation procedure and obtain the maximal and minimal values of spectral causal effects in both directions $S_{1\to2}^{\max}$ and $S_{2\to1}^{\max}$. Then, we compute histograms of these estimates (Fig.S17) and their 99.5-percentiles corresponding to the significance level of p = 0.005. Finally, we compare them with the values of $S_{1\to2}^{\max}$ and $S_{2\to1}^{\max}$ obtained from the actual climate data. For the first kind of surrogate data, we take the optimal AR model (1), set its coupling coefficients to zero $b_{1,k} = b_{2,k} = 0$, and generate time series of the length $N = 1776$ for different time realizations of Gaussian white noises in (1). For the second kind, we take the usual Fourier transform surrogates, where the amplitude of each Fourier component is the same as that for the corresponding component of the climate time series while its phase is taken independently from a uniform distribution over the interval $[0,2\pi]$. In the latter case, one preserves individual power spectra of both climate time series, but removes any statistical dependency between them.

We have obtained that the spectral effect for the climate data $S_{2\to1}^{\max} = 1.4$ strongly (many times) exceeds the 99.5-percentiles $S_{2\to1}^{(99.5)} \approx 0.075$ for the first kind of surrogates and $S_{2\to1}^{(99.5)} \approx 0.29$ for the second kind. Similarly, $S_{1\to2}^{\max} = 1.1$ strongly exceeds $S_{1\to2}^{(99.5)} \approx 0.08$ for the first kind and $S_{1\to2}^{(99.5)} \approx 0.3$ for the second kind of surrogates. According to these results, the obtained non-zero spectral causal effects are statistically significant at least at the p-level of 0.005 and, in fact, at much smaller p-levels.

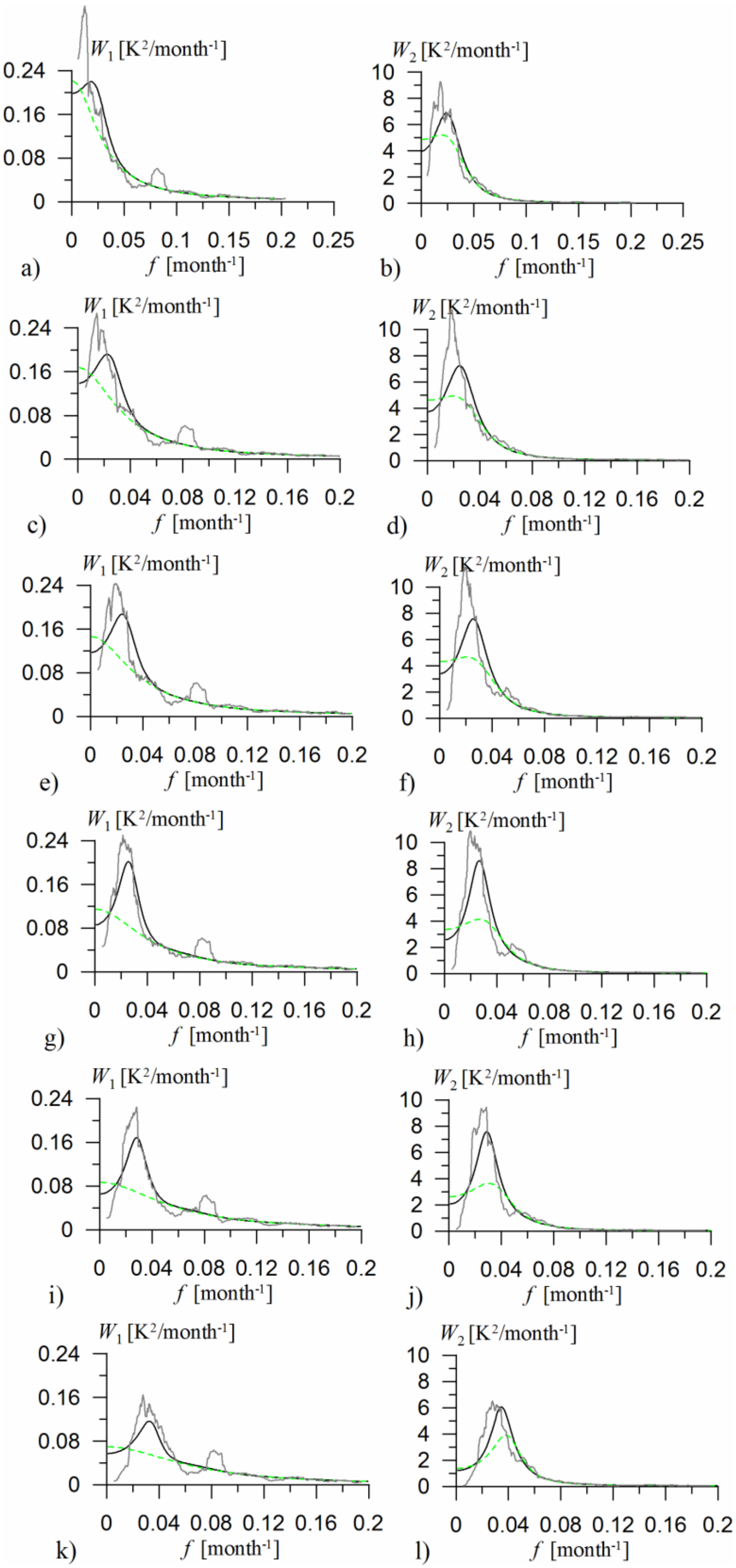

Fig. S14. Power spectral densities for the indices of the NATV (a,c,e,g,i,k) and the ENSO (b,d,f,h,j,l) according to the full AR models (1) (black solid lines) and to the uncoupled AR models (green dashed lines) for different window lengths M: (a,b) 10 years; (c,d) 7 years; (e,f) 6 years; (g,h) 5 years; (i,j) 4 years; (k,l) 3 years. Thin grey lines show smoothed periodograms for the data under analysis.

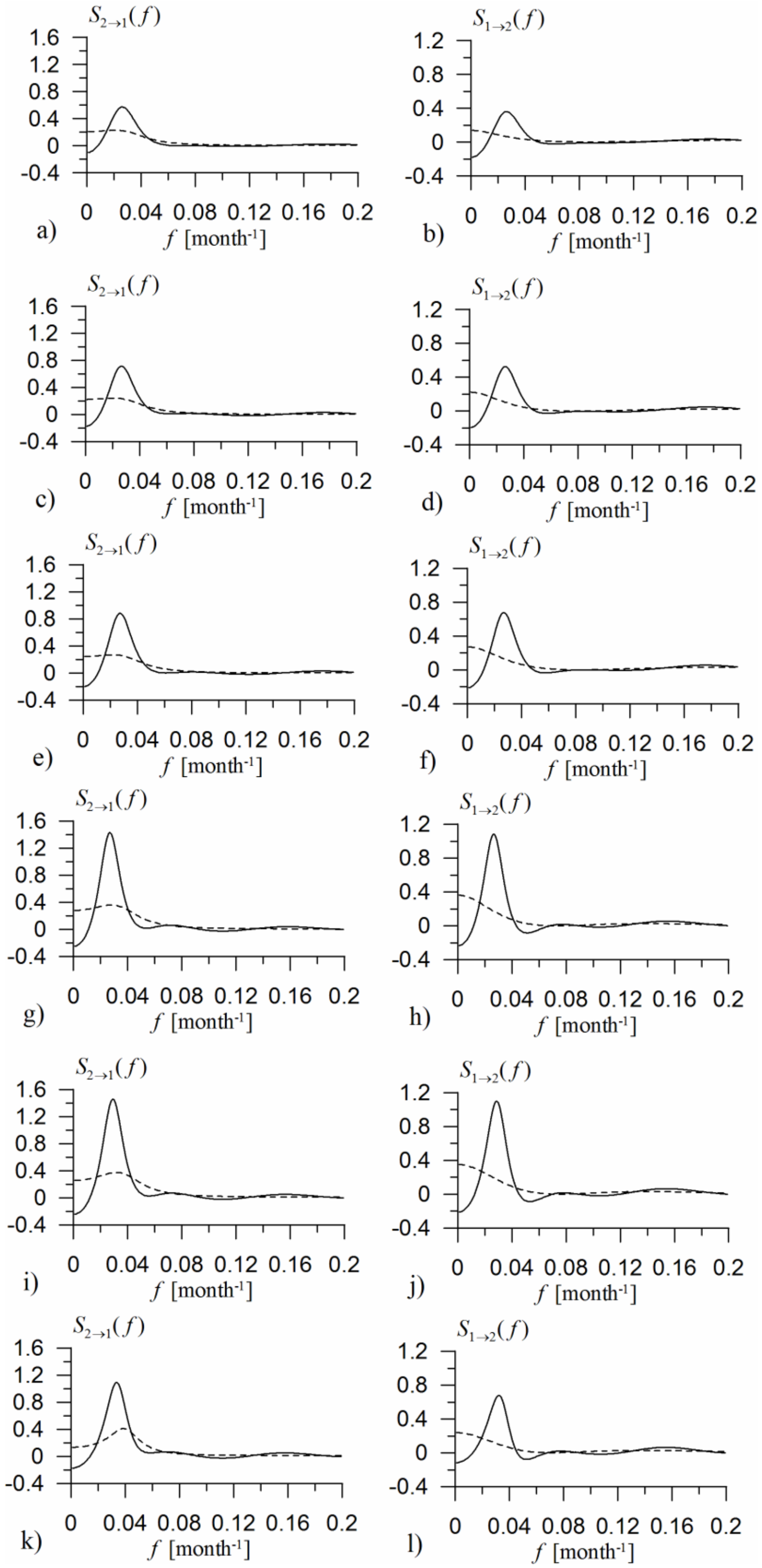

Fig. S15. Spectral DCEs estimated from the time series of the indices of NATV and ENSO according to the full AR models (1) (solid lines) and to the unidirectionally coupled AR models (dashed lines) in the direction ENSO → NATV (a,c,e,g,i,k) and NATV → El-Niño (b,d,f,h,j,l) for different filter window lengths M: (a,b) 10 years; (c,d) 7 years; (e,f) 6 years; (g,h) 5 years; (i,j) 4 years; (k,l) 3 years.

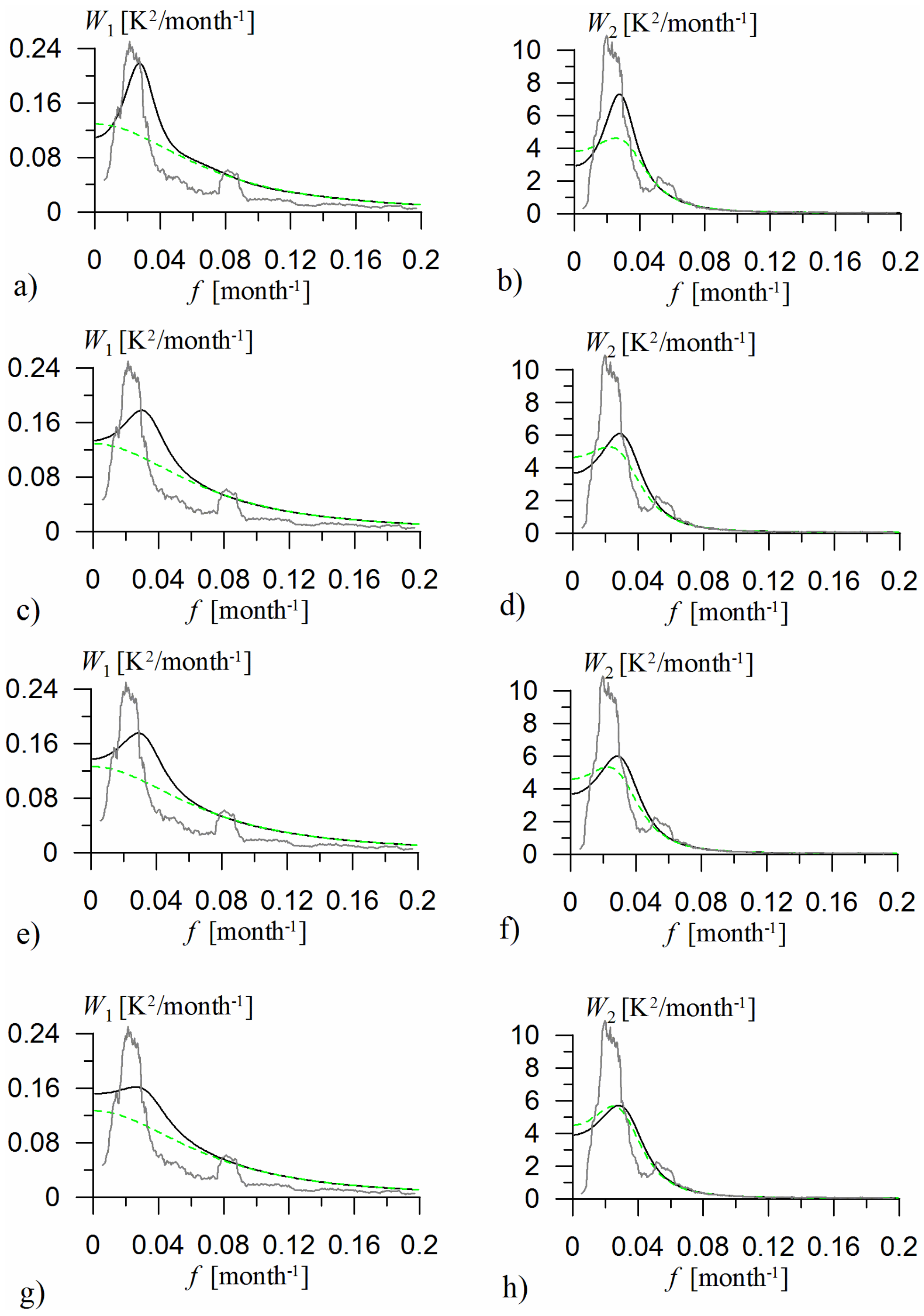

Fig. S16. Power spectral densities for the indices of the NATV according to the ERSSTv5 data (a,c,e,g) and the ENSO (b,d,f,h) according to the full AR models (1) (black solid lines) and the uncoupled AR models (green dashed lines) for different $d_{1\to2}$: (a,b) $d_{1\to2} = 12$; (c,d) $d_{1\to2} = 10$; (e,f) $d_{1\to2} = 5$; (g,h) $d_{1\to2} = 1$. Thin grey lines show smoothed periodograms for those indices.

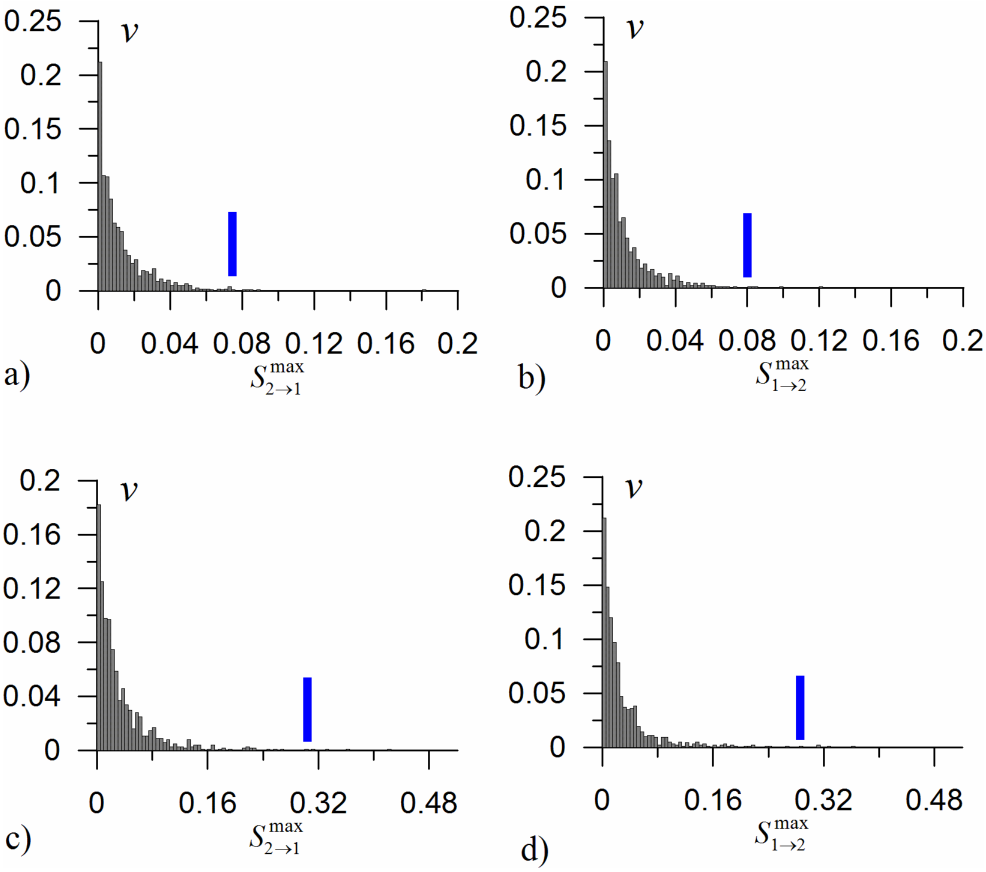

Fig. S17. Histograms for the spectral causal effect estimates obtained from 1000 pairs of surrogate time series: (a,b) empirical AR model (1) with zero coupling coefficients; (c,d) Fourier transform surrogate data with uniformly distributed phases. Blue rectangles indicate 99.5-percentiles. The ordinate *v* denotes the frequency of the estimated values in a bin, bins are shown with grey rectangles.

S3. Directional coupling estimates for three different ENSO indices

These estimates are obtained here both for the AR model dimensions optimized separately for each ENSO index and for a fixed set of the AR model dimensions. Estimates with the usage of the ENSO index I_{N4} are obtained for the optimal AR model (1) with $d_1 = 1, d_2 = 6, d_{2\to1} = 3, d_{1\to2} = 6$. Spectral DCEs in both directions are considerably smaller than those for the two other ENSO indices (Table S5), e.g., the maximal spectral effects are $S_{2\to1}^{max} = 1.0$ and $S_{1\to2}^{max} = 0.6$, the DCEs with respect to the spectral maximum sharpness are $R_{2\to1} = 0.6$ and $R_{1\to2} = 0.6$. Still, the spectral peak at a non-zero frequency for the signal I_{N4} in the AR model disappears under switching the coupling off, i.e. the role of the bidirectional coupling in maintaining the 3-4-years periodicity of the ENSO is even stronger for the index I_{N4}.

The numerical values of the spectral DCEs under the usage of the index I_{N4} are less than those for the index I_{N34}. One could try to explain them by the lower order of the optimal AR model. To check whether this is the case, we present for comparison the last three columns of Table 1 where the AR models with the same set of dimensions are used (each dimension is equal to the maximum value

of this dimension over all three optimal models for the three different ENSO indices) to estimate different DCEs of interest. The comparison between the different ENSO indices for the AR models with the fixed set of dimensions gives the same results as the comparison according to the optimal AR models. Again, the strongest DCEs are obtained for the index Niño-3, somewhat weaker ones for the index Niño-3,4 and the weakest ones for the index Niño-4.

Table S5. Characteristics of the AR models for three ENSO indices. The columns 2-4 are shown for the optimal AR models (indicated by O), the columns 5-7 for a fixed set of the model dimensions.

Characteristics	I_{N3} O	I_{N34} O	I_{N4} O	I_{N3}	I_{N34}	I_{N4}
d_1	1	1	1	2	2	2
d_2	6	6	6	6	6	6
$d_{2\to1}$	5	3	3	8	8	8
$d_{1\to2}$	12	12	6	12	12	12
$G_{2\to1}$, %	4.4	4.3	4.1	4.7	4.6	4.3
$d_{2\to1}p$	$8\cdot10^{-16}$	$9\cdot10^{-16}$	$6\cdot10^{-16}$	10^{-15}	$3\cdot10^{-14}$	$5\cdot10^{-14}$
$G_{1\to2}$, %	3.2	2.3	1.7	3.2	2.3	2.0
$d_{1\to2}p$	$6\cdot10^{-9}$	$4\cdot10^{-6}$	10^{-5}	$6\cdot10^{-9}$	$4\cdot10^{-6}$	$7\cdot10^{-5}$
$S_{2\to1}^{\max}$	1.6	1.4	1.0	2.0	1.9	1.4
$S_{2\to1}^{\min}$	-0.3	-0.3	-0.1	-0.2	-0.2	-0.2
$R_{2\to1}$	1.7-0.0	1.3-0.0	0.6-0.0	2.2-0.0	1.9-0.0	1.4-0.0
$S_{1\to2}^{\max}$	1.3	1.1	0.6	1.4	1.2	1.1
$S_{1\to2}^{\min}$	-0.3	-0.2	-0.3	-0.3	-0.3	-0.3
$R_{1\to2}$	2.9-0.2	2.3-0.2	0.6-0.0	3.5-0.2	3.0-0.2	1.4-0.0

To estimate statistical significance of the differences between the results for the three ENSO indices, we note that the prediction improvement $G_{j\to k}$ = 0.15 % is significantly different from zero at the level of about p = 0.05 for the time series length N = 143 years at hand and small AR model dimensions. Hence, if the difference between the prediction improvements exceeds approximately 0.3 %, it can be regarded roughly as statistically significant at the level of p = 0.05. Then, according to the prediction improvement in the direction ENSO $\to$ NATV the difference between the results for the indices Niño-3 and Niño-3,4 is insignificant, while this difference between the index Niño-4 and any of the two other indices is significant. According to the prediction improvement in the opposite direction, the results for all three indices are significantly different from each other, in particular, the prediction improvement for the index Niño-3 exceeds that for the index Niño-3,4 by 1 %.

According to the spectral DCEs, the comparison results are the same whether one uses optimal AR models or the fixed set of model dimensions. The ordering of the three ENSO indices with respect to the values of the spectral DCEs is the same as that with respect to the prediction improvements. However, it is difficult to estimate statistical significance of the differences between the spectral DCE estimates for the different ENSO indices. To summarize, the qualitative conclusion remains the same: an intradecadal (interannual) spectral peak for each of the two processes under study exists essentially due to the presence of the bidirectional coupling between them; the directional coupling effects are strongest for the region Niño-3, somewhat weaker for the region Niño-3,4, and the weakest for the region Niño-4.

S4. Temporal stability of the directional coupling estimates: 1870-1945 versus 1946-2022

The DCE estimates are also obtained for two different halves of the entire observation interval, i.e. for the periods 1870-1945 and 1946-2022, for all three ENSO indices. So, 6 different bivariate AR models are fitted and provide the corresponding spectral DCE values. The AR model dimensions are optimized separately for each ENSO index and each period to preserve the same procedure for all 6 cases. For another comparison, all DCE estimates are also obtained for a fixed set of the AR model dimensions, each of them being selected to be the largest among the 6 corresponding optimal values. Both approaches give very similar results, so only results of the former one (i.e. that with separately optimized model dimensions) is described here in detail.

The results of the DCE estimation for the two halves are qualitatively similar to the results for the entire observation period (Tables S6 – S8). In particular, spectral causal effects are always considerable and have comparable magnitudes for different periods, i.e. the conclusion about the important spectral role of the bidirectional coupling is robust across subsequent time epochs. Similarly, the Wiener – Granger causality and DCE on stationary variance are always significant and also have comparable magnitudes for different periods.

As for the quantitative comparison, one can make the following conclusions:

For the region Niño-3, almost all DCEs in both directions rise under the transition from the first half (1870-1945) to the second half (1946-2022) of the data under study (Table S6).

For the region Niño-3,4, some DCEs rise ($G_{2\to1}, C_{2\to1}$) while the others either decrease ($G_{1\to2}, R_{2\to1}, S_{1\to2}^{\max}, R_{1\to2}, C_{1\to2}$) or remain almost the same ($S_{2\to1}^{\max}$) under the transition from the first half to the second one (Table S7). So, overall the DCE effects remain roughly the same.

For the region Niño-4, almost all DCEs decrease under the transition from the first half to the second one (Table S8).

Thus, the temporal changes of the estimated DCEs are mutually opposite for the regions Niño-3 and Niño-4, while they are mixed for the “mixed” region Niño-3,4. The latter seems quite reasonable. Still, statistical significance of the conclusions about those non-zero temporal changes is not clear and requires further investigation. Currently, it is just a rough preliminary result at variance with much more reliable conclusion about the important spectral role (large spectral effects) of the bidirectional coupling between the NATV and ENSO.

Table S6. Characteristics of the AR models for the ENSO index I_{N3} for different time epochs and the corresponding optimal AR models whose dimensions are shown in rows 2-5.

Characteristics	1870-2022	1870-1945	1946-2022
d_1	1	1	2
d_2	6	6	5
$d_{2\to1}$	5	2	8
$d_{1\to2}$	12	11	12
$G_{2\to1}$, %	4.4	4.2	6.0
$d_{2\to1}p$	$8\cdot10^{-16}$	10^{-8}	$3\cdot10^{-9}$
$G_{1\to2}$, %	3.2	3.0	4.2
$d_{1\to2}p$	$6\cdot10^{-9}$	0.002	$3\cdot10^{-5}$
$S_{2\to1}^{\max}$	1.6	1.4	2.7
$S_{2\to1}^{\min}$	-0.3	-0.3	-0.3
$R_{2\to1}$	1.7-0.0	1.4-0.0	3.4-0.0
$S_{1\to2}^{\max}$	1.3	1.1	1.9
$S_{1\to2}^{\min}$	-0.3	-0.3	-0.4
$R_{1\to2}$	2.9-0.2	2.0-0.04	4.3-0.2
$C_{2\to1}$	0.26	0.22	0.33
$C_{1\to2}$	0.28	0.23	0.14

Table S7. Characteristics of the AR models for the ENSO index I_{N34} for different time epochs and the corresponding optimal AR models whose dimensions are shown in rows 2-5.

Characteristics	1870-2022	1870-1945	1946-2022
d_1	1	1	2
d_2	6	6	5
$d_{2\to1}$	3	2	8
$d_{1\to2}$	12	11	3
$G_{2\to1}$, %	4.3	4.6	6.0
$d_{2\to1}p$	$9\cdot10^{-16}$	$2\cdot10^{-9}$	$4\cdot10^{-8}$
$G_{1\to2}$, %	2.3	2.5	1.6
$d_{1\to2}p$	$4\cdot10^{-6}$	$8\cdot10^{-3}$	$3\cdot10^{-3}$
$S_{2\to1}^{\max}$	1.4	1.5	1.4
$S_{2\to1}^{\min}$	-0.25	-0.2	0.1
$R_{2\to1}$	1.3-0.0	1.5-0.0	0.8-0.0
$S_{1\to2}^{\max}$	1.1	1.1	0.6
$S_{1\to2}^{\min}$	-0.2	-0.3	-0.3
$R_{1\to2}$	2.3-0.2	1.9-0.0	1.3-0.1
$C_{2\to1}$	0.24	0.26	0.34
$C_{1\to2}$	0.26	0.22	0.12

Table S8. Characteristics of the AR models for the ENSO index I_{N4} for different time epochs and the corresponding optimal AR models whose dimensions are shown in rows 2-5.

Characteristics	1870-2022	1870-1945	1946-2022
d_1	1	1	2
d_2	6	2	4
$d_{2\to1}$	3	3	3
$d_{1\to2}$	6	6	3
$G_{2\to1}$, %	4.1	5.2	3.7
$d_{2\to1}p$	$6\cdot10^{-16}$	$8\cdot10^{-10}$	$5\cdot10^{-7}$
$G_{1\to2}$, %	1.7	4.0	1.8
$d_{1\to2}p$	10^{-5}	$2\cdot10^{-6}$	0.001
$S_{2\to1}^{\max}$	1.0	1.3	0.7
$S_{2\to1}^{\min}$	-0.1	-0.0	-0.0
$R_{2\to1}$	0.6-0.0	0.9-0.0	0.4-0.0
$S_{1\to2}^{\max}$	0.6	1.0	0.4
$S_{1\to2}^{\min}$	-0.3	-0.5	-0.2
$R_{1\to2}$	0.6-0.0	0.3-0.0	0.3-0.0
$C_{2\to1}$	0.23	0.32	0.21
$C_{1\to2}$	0.12	0.05	0.08
